# Patterns and driving factors of functional traits of desert species with different elevational distributions in the Tibetan Plateau and adjacent areas

**DOI:** 10.1186/s12870-024-05080-x

**Published:** 2024-05-09

**Authors:** Ya Hu, Xiangyun Li, Shaokun Wang, Peng Lv, Ping Yue, Min Chen, Xiaoan Zuo

**Affiliations:** 1 Urat Desert-grassland Research Station, Northwest Institute of Eco-Environment and Resources, Chinese Academy of Sciences, Lanzhou, 730000 China; 2 Key Laboratory of Stress Physiology and Ecology in Cold and Arid Regions, Lanzhou, 730000 Gansu Province China

**Keywords:** Functional trait, Elevation, Intraspecific trait variation, Tibetan Plateau

## Abstract

Variations in functional traits serve as measures of plants’ ability to adapt to environment. Exploring the patterns of functional traits of desert plants along elevational gradients is helpful to understand the responses and adaptation strategies of species to changing environments. However, it is unknown whether the relationship between functional traits and elevation is affected by differences in the species’ elevational distributions (elevation preference and species’ range). Importantly, most researches have concerned with differences in mean trait values and ignored intraspecific trait variation. Here, we measured functional traits of desert plants along a wide elevational gradient in the Tibetan Plateau and adjacent areas and explored functional trait patterns over elevation in species with different elevational distributions. We decomposed trait variation and further investigated characterizations of intraspecific variation. Ultimately, the main drivers of trait variation were identified using redundancy analysis. We found that species’ elevational distributions significantly influenced the relationship of functional traits such as plant height, leaf dry matter content, leaf thickness, leaf nitrogen and carbon content with elevation. Species with a lower elevational preference showed greater trait variation than species with a higher elevational preference, suggesting that species that prefer high elevation are more conservative facing environmental changes. We provide evidence that interspecific trait variation in leaf thickness and leaf carbon content decreased with increasing species’ range, indicating that increased variations in resistance traits within species make greater responsiveness to environmental changes, enabling species a wider range. Elevation, temperature and precipitation were the main drivers of trait variation in species with a low elevational preference, while the effect of precipitation on trait variation in species with a high elevational preference was not significant. This study sheds new insights on how plants with different elevational distributions regulate their ecological strategies to cope with changing environments.

**Supplementary Information:**

The online version contains supplementary material available at 10.1186/s12870-024-05080-x.

## Introduction

Predicting how different specie will respond to environmental changes is challenging due to the diversity of natural ecosystems [1]. Functional traits provide a method for disentangling community responses to environmental changes by linking environment with individual performance [2–4]. Functional traits are measurable characteristics of an individual that represent species adaptive responses to abiotic and biotic factors [5, 6]. There has been a classic problem in ecology on what cause functional trait variations along climate gradients [4, 7]. This knowledge is crucial for foreseeing how climate changes will affect species, species interactions, and ecosystem functioning [8]. The distribution of functional traits along environmental gradients is explained by mechanisms relating to physiological constraints on species [9]. Physiology-based theories presuppose that changes in the physical environment and physiological restraints control the distribution and evolution of organisms, with implications for the distribution of morphological traits [8].

Elevational gradients offer suitable environments for enhancing inference of the mechanical causes of ecosystem functioning due to their varied environmental and climatic circumstances [6]. Some functional traits, particularly those that relate to plant height, leaf size and resource acquisition, are strong predictors of plant performance, differ between species, and can be used to infer changes in ecosystem functioning at broad ecological scales [10, 11]. For example, with increasing elevation, leaves get thicker and smaller [4]. In harsher situations, plant height and leaf size tend to decline, whereas leaf nutrient contents vary with leaf morphology, elevation, and climatic conditions [12]. The variations in functional trait over elevational gradients are expected to explain plant ecological strategies [13].

Trait-based ecology has, up to this point, mostly emphasized the differences in traits between species [2, 14, 15]. However, there is mounting evidence that intraspecific variation, rather than interspecific variations, contributes more to trait variation caused by environmental factors [1, 16, 17]. Intraspecific variation accounts for about a quarter of total trait variation globally [17], but this proportion is predicted to increase in harsh environments due to the filtering effect of environment on the trait expression [18]. Large intraspecific trait variation may conceal or change the relationships among interspecific traits, limiting the usefulness of interspecific variation for ecological prediction at different scales [19]. Therefore, investigating intraspecific trait variations may provide a more comprehensive answer to community construction and ecosystem function maintenance [17].

The Tibetan Plateau region is characterized by high average elevations and wide elevational gradients, due to the topographic features and the atmospheric circulation characteristics, harboring not only unique alpine ecosystems, but also a variety of natural ecosystem types such as forests, meadows, steppes and deserts appear from southeast to northwest [20, 21]. Therefore, the Tibetan Plateau has nurtured many unique plant resources with high biological and genetic research values, which are important for biodiversity maintenance and biological resource conservation [21, 22]. The Tibetan Plateau is not only an important ecological security barrier, but also a sensitive and fragile zone to global climate changes. As the global climate change process advances, glacial retreat, permafrost melting and desertification are becoming more prominent, accelerating the degradation of vegetation in the Tibetan Plateau [23]. Exploring the status of desert plants on the Tibetan Plateau can provide a theoretical basis for desertification control, biological resource conservation and sustainable development of the ecosystem.

Plants respond differently to different elevational gradients, but there are fewer studies on the response of traits to different elevational distribution ranges. Interspecific and intraspecific trait variations are major components of plant functional trait variation, but intraspecific trait variations across a large elevational gradient merits further research [2, 24]. The objectives of the present study were: (i) to establish the relationship between functional traits and elevation in desert species with different elevational preferences and elevational distribution ranges; (ii) to explore the sources of variation of functional traits and to determine the proportion of intraspecific variation and (iii) to identify the main environmental factors that influence functional traits of species with different elevational distributions.

## Materials and methods

### Study area

According to comprehensive natural geographic zoning, distribution of deserts and desertification in China and land use conditions, we selected a typical desert ecosystem of the Tibetan Plateau and adjacent areas as our study area. The region reaches an average elevation of 4000 m a.s.l., and nearly a quarter of its northwestern area is alpine, with altitudes above 5000 m a.s.l. The moisture status of the study area has large differences, with annual precipitation mostly below 900 mm, decreasing from east to west and from south to north. The sampling sites were selected in the desert ecosystems of the Tibetan Plateau and adjacent areas, ranging from 813 to 5930 m a.s.l.

### Sampling and trait measurements

A total of 414 study sites were selected in the desert ecosystems of the Tibetan Plateau and adjacent areas, and vegetation surveys were conducted over 4 years (Table S1). At each study site, typical and representative plant communities were selected and a 100 m × 100 m sampling area was established. Within the sampling area, five 10 m×10 m shrub sampling plots and nine 1 m×1 m grass sampling plots were set up to investigate the species composition in shrub and grass sampling plots, respectively.

For dominant species of the community, 6 functional traits were measured based on the relevance to plant survival strategies and the feasibility of field measurements [10, 25], including plant height, specific leaf area (SLA), leaf dry matter content (LDMC), leaf thickness (LT), leaf carbon content (LCC) and leaf nitrogen content (LNC). Plant height of each species was measured at the same time as the vegetation survey distancing from soil to highest leaf. We selected 5–10 individuals and at least 10 leaves from each dominant species within a site for determination of functional traits. The SLA, LDMC and LT were measured referring to standard protocols [25]. The leaves were dried and crushed for the determination of LCC and LNC by elemental analyzer (Costech, Milano, Italy).

### Elevational distribution indicators

The elevational preference (EP) and species’ range (SR) can be used to explain two aspects of elevational distributions, reflecting the species’ preference for habitat elevation and the range over which the species can be distributed, respectively [1]. A species’ EP represents its median elevation in relation to all species, calculating by the following formula. EP ranges from 0 to 1, with values close to 0 for species with median elevation that is near to the lower elevation and 1 for species with median elevation that is near to the higher elevation.

$${Elevational\, preference}_{i}=1+\left[\frac{{Ele\left(Med\right)}_{i}-Ele\left(Max\right)}{Ele\left(Max\right)-Ele\left(Min\right)}\right]$$

Where ‘Elevational preference_i_’ is the elevational preference of species i, ‘Ele (Med)_i_’ is the median elevation of species i, ‘Ele (Max)’ is the maximum elevation of all species and ‘Ele (Min)’ is the minimum elevation of all species.

We calculated each target species’ SR, which reflects its elevational distribution in comparison to all species. SR ranges between 0 and 1 with values near to 0 for species with narrower elevation ranges, and 1 for species with wider elevational ranges. We estimated the SR by the following equation.

$${Species\, range}_{i}=\left[\frac{{Ele\left(Max\right)}_{i}-{Ele\left(Min\right)}_{i}}{Ele\left(Max\right)-Ele\left(Min\right)}\right]$$

Where ‘Species range_i_’ is the distribution range of species i, ‘Ele (Max)_i_’ is the maximum elevation of species i and ‘Ele (Min)_i_’ is the minimum elevation of species i.

### Statistical analysis

To examine the patterns of plant trait variation in desert species with different elevational distributions over elevation, we constructed mixed-effects models for plant height, SLA, LDMC, LT, LNC and LCC using the lmer function from the lme4 package.

By fitting linear mixed effects models with a fixed intercept and random effects for region, site, functional group and species, we quantified the amount of trait variation for each species and trait at each nested scale using variance decomposition. The random effect variances in this equation stand in for variance between regions, sites, functional groups and species, whereas the residual variance represents samples within species (intraspecific trait variation).

We ran a redundancy analysis (RDA) on all trait measurements and elevation, temperature, precipitation, soil pH, soil electrical conductivity (EC), soil clay content (Clay), soil sand content (Sand), soil nitrogen content (SNC) and soil carbon content (SCC) to determine the relationships between functional traits and environmental factors. RDA was analyzed by the rda function form the vegan package. All the data analysis was carried out using R (R Development Core Team 2022).

## Results

### Patterns of desert plant traits along elevational gradients

At the overall level, plant height, LT, LNC and LCC gradually decreased with increasing elevation (Figure S2). In detail, height showed a decreasing overall pattern with elevation in the responses of most species, meaning higher elevations resulted in shorter plants. Although trends varied widely among species, more than half of the species showed a decreasing trend, resulting in a significant decline in LT and LCC with elevation. However, there were few species with a decreasing trend, but overall LNC decreased significantly along elevation, probably due to interspecific differences. Moreover, Differences between species trends may account for the non-significant relationship between SLA, LDMC and elevation (Figure S3-S8).

### Relationships between functional traits and elevation for species with different elevational distributions

**Fig. 1:**
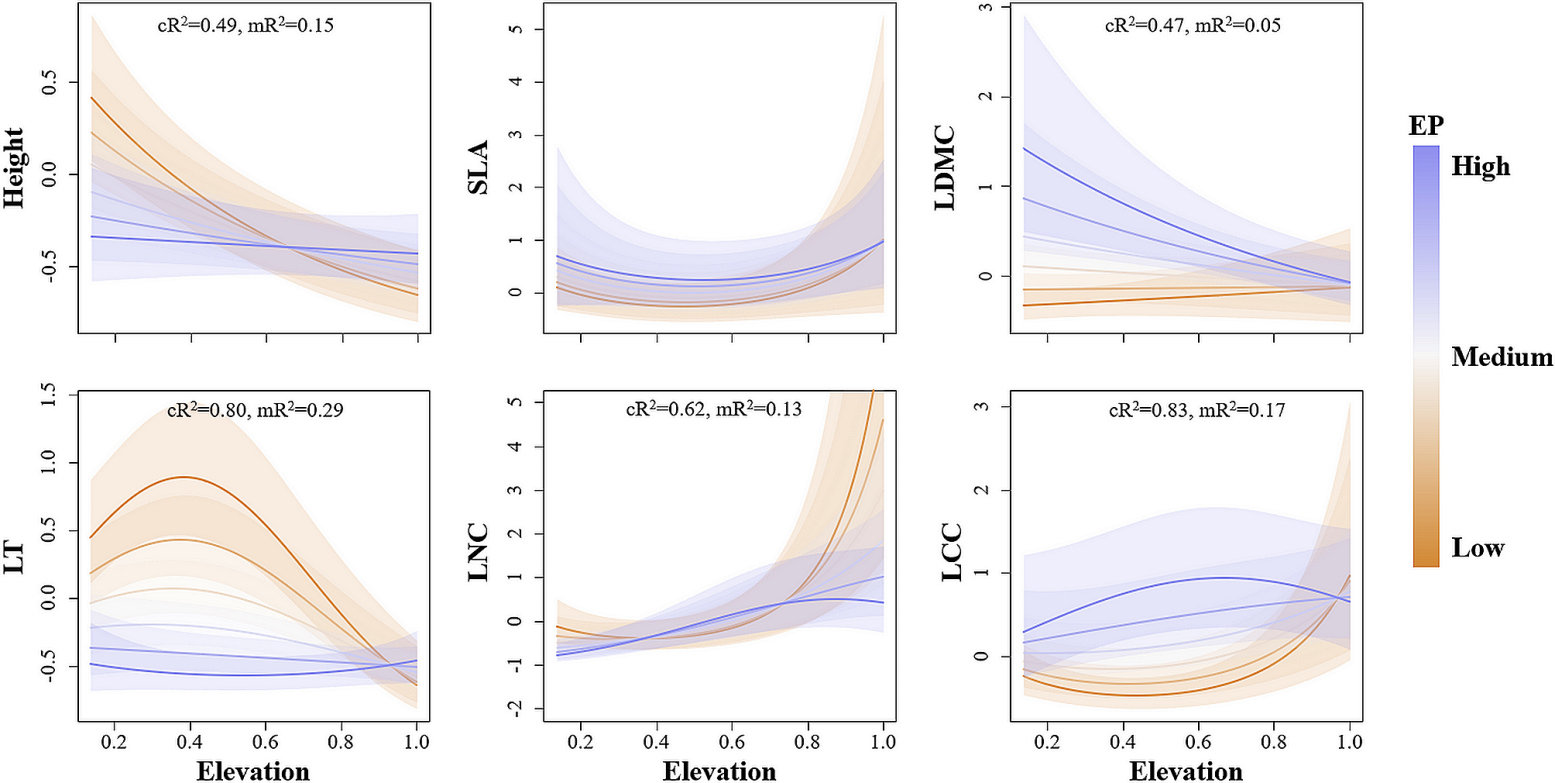
Relationship between functional traits and elevation, as influenced by species elevational preference (EP). Functional traits: Plant height, specific leaf area (SLA), leaf dry matter content (LDMC), leaf thickness (LT), leaf nitrogen content (LNC) and leaf carbon content (LCC). Shade areas are the 95% confidence intervals. cR^2^ represents conditional R^2^ value, and mR^2^ represents marginal R^2^ value. Trait values and elevation were standardized

Elevation distributions, namely EP and SR, largely influences the relationship between functional traits and elevation. There were significant interactions between elevation and EP for traits such as plant height, LDMC, LT, LNC and LCC. Plant height, LT, and LNC varied less along the elevational gradients in high EP species, whereas low EP plants showed greater variation in functional trait values (Fig. 1). Among them, plant height and LT of low EP species decreased at higher elevations, while LNC increased. On the contrary, LDMC of high EP species decreased gradually along the elevation, while that of low EP species remained at a low level. In addition, LCC was highest and lowest in median elevation for high and low EP species, respectively.

**Fig. 2:**
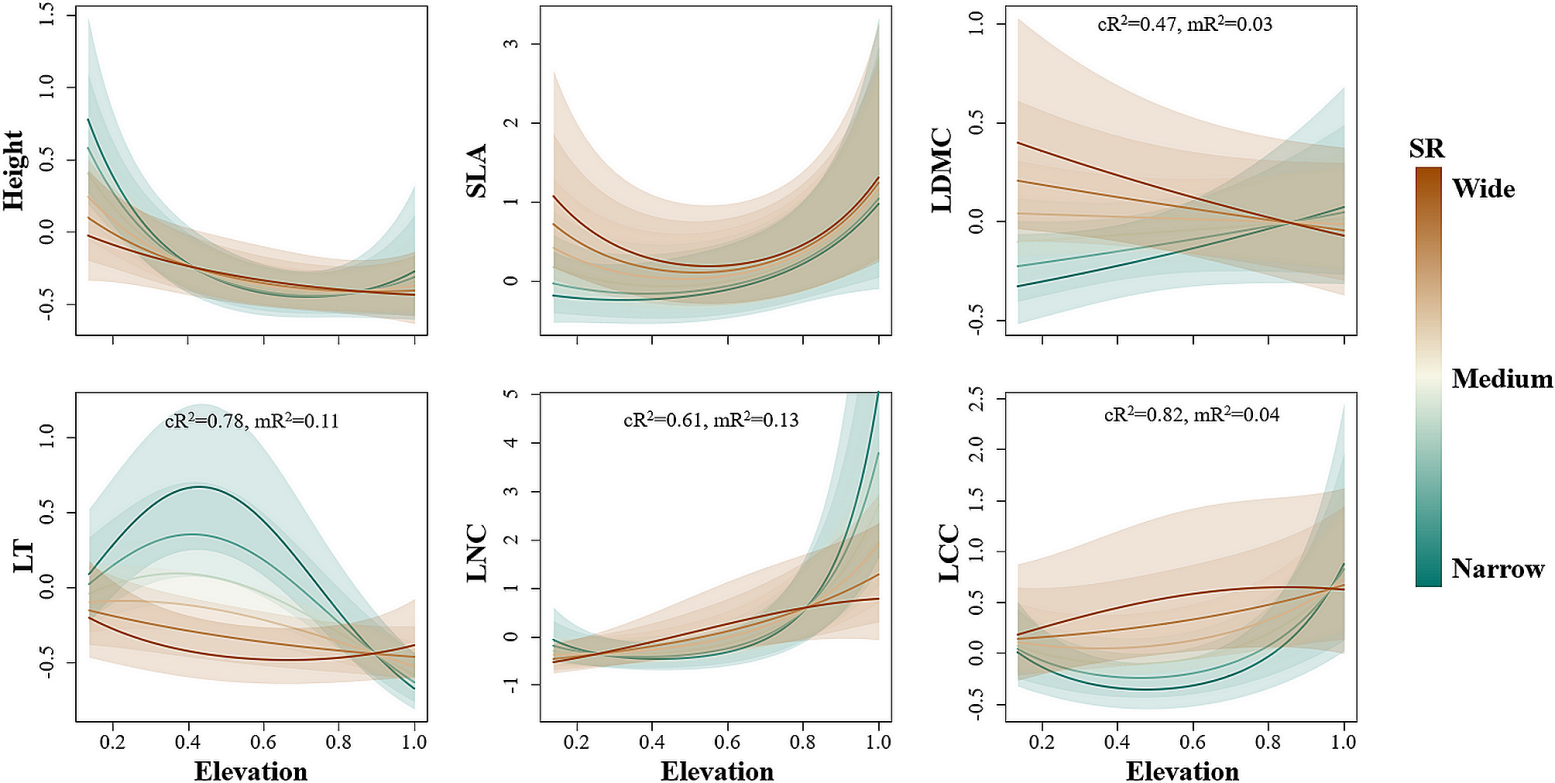
Relationship between functional traits and elevation, as influenced by species’ elevational range (SR). Functional traits: Plant height, specific leaf area (SLA), leaf dry matter content (LDMC), leaf thickness (LT), leaf nitrogen content (LNC) and leaf carbon content (LCC). Shade areas are the 95% confidence intervals. cR^2^ represents conditional R^2^ value, and mR^2^ represents marginal R^2^ value. Trait values and elevation were standardized

The interactions of elevation and SR had significant effects on LDMC, LT, LNC and LCC. LT, LNC and LCC varied less along the elevational gradients in wide SR species, whereas narrow SR plants displayed a greater variation in functional trait values (Fig. 2). Wide and narrow species had opposite trends in LDMC. As the elevation increased, the LDMC of wide SR species decreased, and that of narrow SR species increased.

### Species’ elevational distributions

Using EP as the horizontal axis and SR as the vertical axis, we plotted elevational distributions of desert species with observations greater than 10 times. The clustering of plant species with similar elevational distribution characteristics could be found in the Figure S9. LN Group had 8 species and was characterized by low EP and narrow SR (0< EP<0.3, 0< SR<0.3), revealing that these species prefer lower elevations and only distributed at lower elevations. All species in this group were shrubs. We divided the 4 species into LW Group with low EP and wide SR (0< EP<0.3, 0.7< SR<1.0), indicating that these species prefer lower elevations but have a broader elevational distribution. This group consisted mainly of shrubs and forbs. Four species were classified into the HN group with high EP and narrow SR (0.7< EP<1.0, 0< SR<0.3), considering that these species were exclusive to high-elevation habitats. In this group, there were only two functional types (forbs and shrubs), and the species with the largest proportion were forbs. Ultimately, We classified 7 species into HW group with high EP and wide SR (0.7< EP<1.0, 0.7< SR<1.0), showing that these species prefer higher elevations but have a broader elevational distribution. In HW group, the most represented species were forbs and graminoids (Figure S9; Table S2).

### Sources of variation in functional traits

**Fig. 3:**
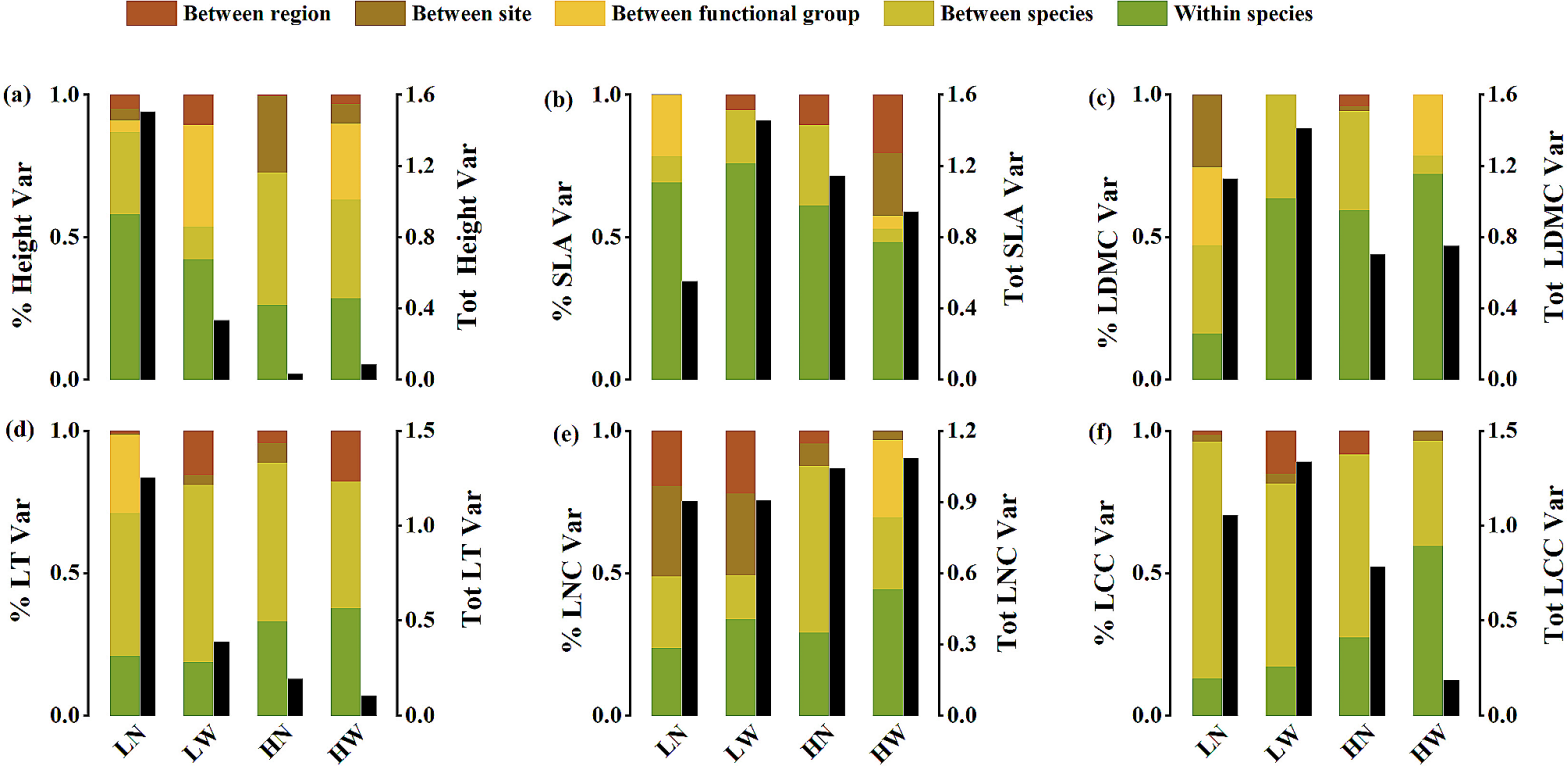
Variance decomposition of height, specific leaf area (SLA), leaf dry matter content (LDMC), leaf thickness (LT), leaf nitrogen content (LNC) and leaf carbon content (LCC) measured across species with different elevational distributions. LN: species with low elevational preference and narrow species’ range; LW: species with low elevational preference and wide species’ range; HN: species with high elevational preference and narrow species’ range; HW: species with high elevational preference and wide species’ range. Colored bars show proportion of total trait variance (‘% trait Var’) while black bar shows absolute amount of variance (‘Tot trait Var’)

We found that differences within species explained trait variation of plant height in low EP desert plants (Fig. 3a). Both total and intraspecific variation in plant height decreased significantly with increasing EP, and the total variation decreased with increasing SR (Figure S10 a; Figure S11 a). The intraspecific variation of SLA and LDMC showed a high proportion of the total trait variation accounting for an average of 63.68% and 52.96% of total variation, respectively, and the total variation in SLA increased with increasing SR (Fig. 3b c; Figure S11 b). LT had large interspecific variation, and total variation in LT decreased significantly with increasing EP and SR (Fig. 3d; Figure S10 d; Figure S11 d). LNC of wide SR species had a large intraspecific variation, and interspecific variation in LNC increased with increasing EP (Fig. 3e; Figure S10 e). Moreover, LCC in HW group exhibited large proportion of intraspecific variation, and interspecific variation in LCC decreased with increasing SR (Fig. 3f; Figure S11 f).

For species presenting these four distributions, we took into account patterns of intraspecific variation over elevation. We found that the significant negative feedback of plant height with elevation was reflected at the level of individual species for most species in all groups except the HW species group (Figure S12 a). SLA and LCC had significant intraspecific trends only for species in the LN and HW groups with the mostly decrease trend (Figure S12 b f). Most species with significant intraspecific variation trends in LDMC showed negative responses to elevation (Figure S12 c). LT of species in the LN group showed more constraint, with half of the species showing an increase trend and a quarter of the species a decrease trend (Figure S12 d). LNC showed the most consistent variance constraint with elevation in HW group, with half of the species showing higher trait values at higher elevation (Figure S12 e).

### Relationships between functional traits and environmental factors

**Fig. 4:**
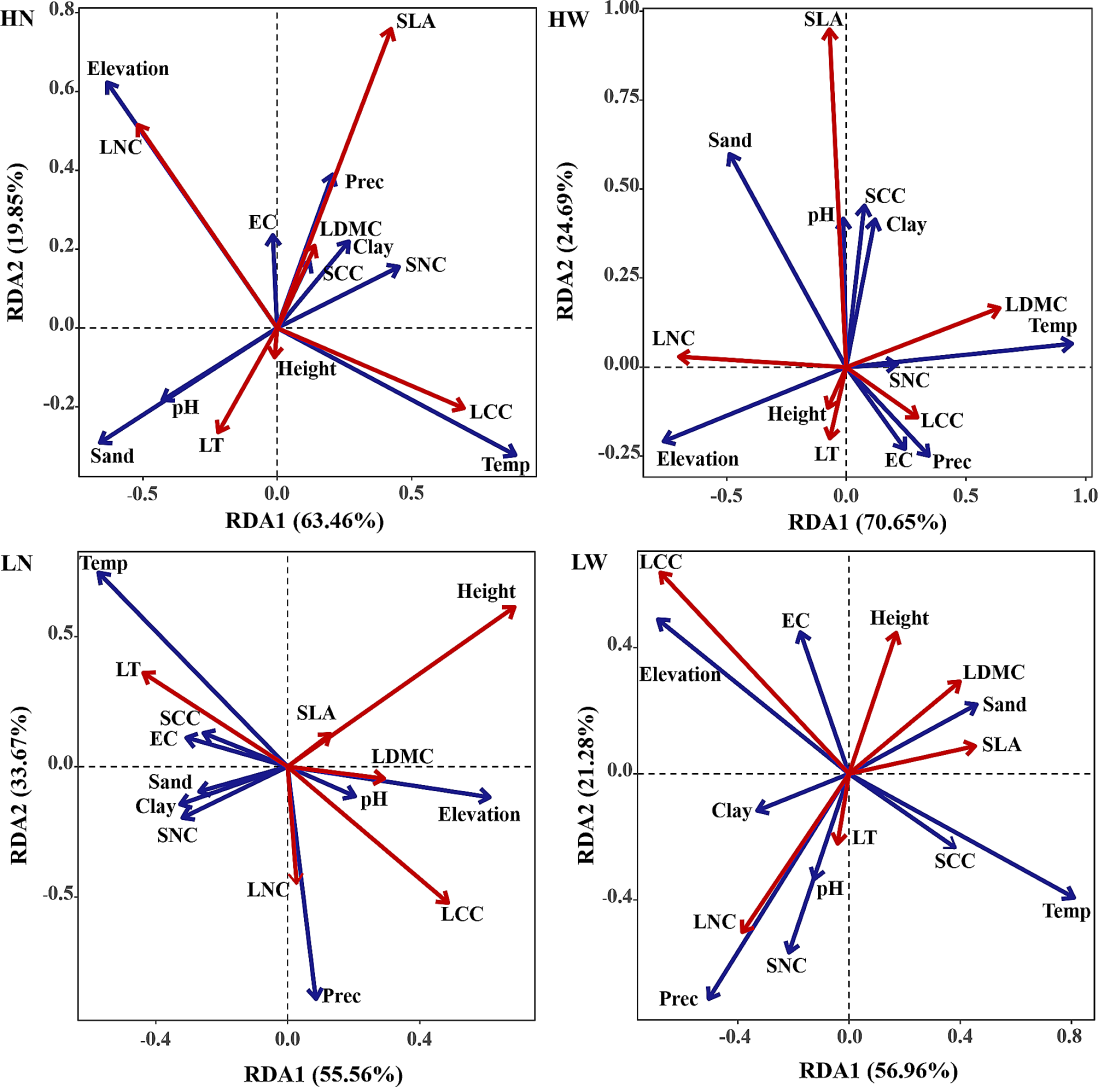
Redundancy analysis of functional traits and environmental factors across species with different elevational distributions. Functional traits: plant height, specific leaf area (SLA), leaf dry matter content (LDMC), leaf thickness (LT), leaf nitrogen content (LNC) and leaf carbon content (LCC). Environmental factors: elevation, annual mean temperature (Temp), annual mean precipitation (Prec), soil pH (pH), soil electrical conductivity (EC), soil clay content (Clay), soil sand content (Sand), soil nitrogen content (SNC) and soil carbon content (SCC). LN: species with low elevational preference and narrow species’ range; LW: species with low elevational preference and wide species’ range; HN: species with high elevational preference and narrow species’ range; HW: species with high elevational preference and wide species’ range. Red lines indicate functional traits, and blue lines indicate environmental factors

The relationship between functional traits of desert plants and environmental factors was further analyzed by RDA analysis, and the results showed that the cumulative explanation rates of the first two axes were 89.23%, 78.24%, 83.31% and 95.34%, respectively (Fig. 4). The first two axes could reflect the relationship well, and were mainly determined by axis I. In detail, temperature, precipitation and elevation provided a better explanation for the variation in functional traits in the LN group (Table S3). Environmental factors explain more about plant height, LCC and LT in the LN group. Temperature, elevation, precipitation, SNC, sand content, SCC and EC significantly affected (*p* < 0.05) the differences in functional traits of desert plants in the LW group. LCC, LNC and height of the LW group were better explained by environmental factors (Fig. 4; Table S3). In the HN group, temperature, elevation, sand content, SNC and pH were determined to be significant environmental factors (*p* < 0.05) affecting SLA, LNC and LCC of desert plants (Table S3). Temperature, elevation and sand content had significant effects on the variation of functional traits in the HW group, especially for SLA, LDMC and LNC.

## Discussion

Our results demonstrated that elevational distributions affected trait shifts. These shifts were manifested in both trait values and variance portioning. The significant value changes in plant height and LT over elevation were mainly driven by species with lower EP, while LDMC was mainly driven by species with higher EP. Higher EP species exhibit lower trait variation compared to lower EP species, which may have a reduced potential to respond to environmental gradients [1]. Moreover, intraspecific trait variations of species with different elevational distributions demonstrated different adaptation strategies corresponding to elevation changes. We provide preliminary evidence that elevation, temperature and precipitation were the main factors influencing functional traits in lower EP species, while higher EP species were not influenced by precipitation.

### Variation in functional traits across a wide elevational gradient

A number of factors, including temperature, precipitation, solar radiation, and atmospheric pressure, can influence the patterns of functional traits along elevational gradients [26]. Plant height, LT, LNC and LCC significantly decreased with increasing elevation. These traits of plant growth and resource utilization indicate a survival strategy of desert plants along a wide elevational gradient. Variations in plant height are supported by previous studies [12, 21]. Long documented, the negative correlation between plant height and elevation has been considered to be linked to community assembly and plastic variation of plants [12, 27]. For example, plant communities at higher elevations have lower height compared to those at lower elevations [28]. Individuals of the same species at higher elevations tend to be shorter than those at lower elevations, according to homogeneous garden trials, indicating intraspecific adaptation of plant size to elevation [12].

Variations in leaf size along elevational gradients are determined by different climatic factors and soil conditions [29]. It is generally accepted that the leaves become smaller and thicker with increasing elevation resulting in low SLA, high LDMC and high LT [4, 25, 30]. This can be explained in terms of both water and heat. Firstly, because leaf size is a key factor in controlling evapotranspiration and is used as a proxy for energy and water balances [29, 31], variations in leaf size caused by rising elevation may be a result of feedback from soil moisture [32]. In this experiment, both LT and SLA were strongly correlated with the precipitation. Secondly, variations in leaf size with elevation probably reflect the divergence in temperature between the day and night [4], with accumulation of matter in leaves [33]. Our results showed that the SLA in the LW group and the LDMC in the HW group were strongly related to temperature, which confirmed this opinion. Consequently, the variations in these traits may reflect the widespread and pervasive role of water-heat exchange in influencing plant plasticity [4]. However, our results suggested that the overall patterns of SLA and LDMC along elevation were not significant. This may be due to the complexity of trait variation within different species. Results from a study that measured SLA for 21 species at different elevational ranges found that SLA increased across elevational gradients [34]. This pattern may indicate that plants in higher elevations have lager photosynthetic capacity to ensure rapid carbon uptake [34, 35].

In line with previous studies, LNC and LCC decreased with increasing elevation mainly due to temperature and precipitation decreased [13, 36]. This result is consistent with the plant temperature-physiological theory, which postulates that plants’ metabolic activity slows down in the cold [12]. Transpiration, along with feedback from the soil and atmosphere, is the primary factor influencing changes in leaf nutrient concentrations [37]. These directional shifts in traits along elevational gradients collectively suggest that plants adjust their height, leaf size and nutrient concentration to make up for decreased photosynthetic capacity in response to lower temperatures, reduced precipitation, rising solar radiation and increased atmospheric pressure [21].

### Species occupying different elevational distributions

Different plants have different elevational preferences, which is partially reflected in their spatial distribution [1]. The EP and SR of desert species were considered as X and Y axes, and we identified four categories of desert plants. Species in LN group characterized by low EP and narrow SR, contain *Alhagi camelorum*, *Anabasis brevifolia*, *Halostachys caspica*, *Haloxylon ammodendron*, *Kalidium foliatum*, *Nitraria tangutorum*, *Tamarix chinensis* and *Zygophyllum xanthoxylum*. These species, mostly drought and salinity tolerant plants, usually have small and tough leaves to conserve water and prevent transpiration [38], thus tending to colonize in specific areas, such as the saline and arid lands. LW group contains drought-tolerant species, and it is also characterized by a high resistance to harsh environments and high reproductive capacity, thus having a relatively wide distribution. Species in HN group with high EP and narrow SR, contain *Asteraceae wellbyi*, *Christolea crassifolia*, *Oxytropis microphylla* and *Stellera chamaejasme*. These species are cold tolerant and grow at higher elevations. Especially, *Asteraceae wellbyi* is endemic to Tibet. Species in the HW group are highly adaptable and barrenness tolerant. The wide distribution may be due to their high vigor and diverse reproduction modes, which enable them to occupy the ground quickly, as well as a well-developed fibrous root system and some physiological and ecological characteristics essential for adaptation to environmental stress [39].

### Trait variation in species with different elevational distributions

For species that inhabit various elevational distributions, we would anticipate different trait values and variation portioning. First of all, we considered functional trait values over elevation for species with different elevational distributions. Our study suggested that the patterns of trait change with elevation depended on the EP and SR. In particular, the main reason for the decrease in plant height and LT with elevation is the species with a low EP. In contrast, elevation-induced LDMC reduction was primarily caused by high EP species. These results suggest that species with different elevational preferences may have different strategies for functional trait variation in response to environmental changes. Desert plants with a high EP may be subject to more abiotic stressors and less interspecific competition than species with a low EP, which may help them stick to their conservative growth strategy of staying small [1]. Desert plants with a low EP tend to be shrubs with succulent leaves that generally have lower LDMC to withstand drought [25, 40]. Most importantly, the inconsistency in the relationship between LDMC and elevation may stem from the species with different SR. In a narrow SR, the changes in LDMC were consistent with most studies [4, 25, 30], suggesting that hydrothermal conditions play an important role in the trait response process [4, 32]. Over a wide SR, however, variation in LDMC may be caused primarily by characteristics of different species. As mentioned before, species of LN group are mainly drought-tolerant shrubs and salt plants, and species of HN group are mainly cold-tolerant graminoids.

Trait variation across elevational gradients may also be a means by which desert species convey their varying preferences for habitat. We offered preliminary proof that desert species with various elevational distributions have diverse patterns of trait variation portioning. According to our results, structural traits of high EP plants show relatively little variation with elevation, which may point to a higher capacity for adapting to environmental changes [1, 34, 41]. While species with a high EP show greater interspecific variation in nutrient trait values with elevation. Given the potential effects of climate change, plant species with relatively high trait variability may be more adaptable to different environmental situations than those with relatively low trait variability [1]. Moreover, intraspecific variations in LNC and LCC were higher in species with a wide SR than in species with a narrow SR, highlighting the high trait plasticity of plant carbon and nitrogen content in widely distributed species. The global mean value of intraspecific variation was 25% [42], and the contribution of intraspecific variation to total trait variation was either equal to or greater than this value for the different groups in this study, despite tough environmental conditions and wide species ranges in Tibetan Plateau. However, intraspecific trait variation varied considerably for different traits, e.g., intraspecific trait variation in SLA and LDMC accounted for about 50% of the total variation, which was consistent with previous studies [30]. Our results provide evidence that the distribution of species along environmental gradients is constrained by intraspecific trait variation [34]. Taken together, the results of this study revealed that, plant establishment and adaption success under varying environmental conditions can be attributed to differences in functional traits [6, 29].

### Response of functional traits to environmental factors

In addition to the influence of the species’ elevational distributions of the plant itself, external environmental factors are important for variation in plant functional traits. Temperature and precipitation are important determinants of the regional climate type and significantly affect plant growth and development. We discovered that species with a high EP faced constraints from elevation and temperature, those with a low EP mostly derived their functional traits from elevation temperature and precipitation. Species with high EP are mostly located in the alpine desert of the Tibetan plateau, where the presence of cold climatic conditions and permafrost prevent plants from efficiently utilizing water [43]. Therefore, changes in the functional traits of desert plants are not significantly influenced by precipitation in this area. Our results suggest that LNC was negatively correlated with temperature, which is consistent with previous studies in field surveys and simulated controlled experiments [44, 45]. This may because that high nitrogen content at low temperatures is needed to compensate for the reduced biochemical efficiency caused by the reduction of high-nitrogen enzyme activity [46]. Alternatively, warmer climate accelerates the plant growth process, thus diluting LNC [45]. Precipitation negatively correlates with SLA for low EP species, suggesting the plant adaptation strategies to maximize carbon income and minimize water consumption under drought stress [4, 47]. In this study, soil properties such as sand content and SNC have important effects on the formation of functional traits in desert plants, as soil is a material and energy source for plant growth and development.

## Conclusions

We discovered that desert plant species displayed different trait trends over elevation, and that these associations relied on the elevational distributions (elevational preferences and species’ ranges) of the individual species. In particular, species with lower elevational preferences expressed higher trait variation in structure trait than those with higher elevational preferences. It was suggested by the increased intraspecific variation of SLA and LDMC that these species may be better adapted to biotic and abiotic changes. Plant species with lower elevational ranges have trait-elevation connections that are widely applicable globally, but LDMC at wider elevational ranges show opposite trends, suggesting that interspecific variation plays an important role in size-related traits at large scales. Most importantly, the main controlling factors of functional traits differed among species with different elevational distributions. Our experiments provide preliminary evidence that desert species with different elevational distributions have different trait distribution patterns and adaptation mechanisms.

### Electronic supplementary material

Below is the link to the electronic supplementary material.

Supplementary Material 1

## Data Availability

Data is provided within the manuscript or supplementary information files.

## Abbreviations

EC: Electrical conductivity
EP: Elevational preference
LCC: Leaf carbon content
LDMC: Leaf dry matter content
LNC: Leaf nitrogen content
LT: Leaf thickness
RDA: Redundancy analysis
SCC: Soil carbon content
SLA: Specific leaf area
SNC: Soil nitrogen content
SR: Species’ range
