# Supplementary material for: Patterns and driving factors of functional traits of desert species with different elevational distributions in the Tibetan Plateau and adjacent areas
Source: BMC Plant Biol. 2024 May 9;24:371. doi: 10.1186/s12870-024-05080-x (PMC11080261; doi:10.1186/s12870-024-05080-x)
Supplement: Supplementary file 1 — Supplementary Material 1 [file 12870_2024_5080_MOESM1_ESM.docx]

**Patterns and driving factors of functional traits of desert species with different elevational distributions in the Tibetan Plateau and adjacent areas**

**Ya Hu ^a,b^, Xiangyun Li ^a,b,c^, Shaokun Wang ^a,b^, Peng Lv ^a,b^, Ping Yue ^a,b^, Min Chen ^a,b^, Xiaoan Zuo ^a,b,*^**

^a^ Urat Desert-grassland Research Station, Northwest Institute of Eco-Environment and Resources, Chinese Academy of Sciences, Lanzhou, 730000, China

^b^ Key Laboratory of Stress Physiology and Ecology in Cold and Arid Regions, Gansu Province, Lanzhou, 730000, China

^*^ Correspondence: zuoxa@lzb.ac.cn

Postal address: Northwest Institute of Eco-Environment and Resources, Chinese Academy of Sciences, Lanzhou, 730000, China


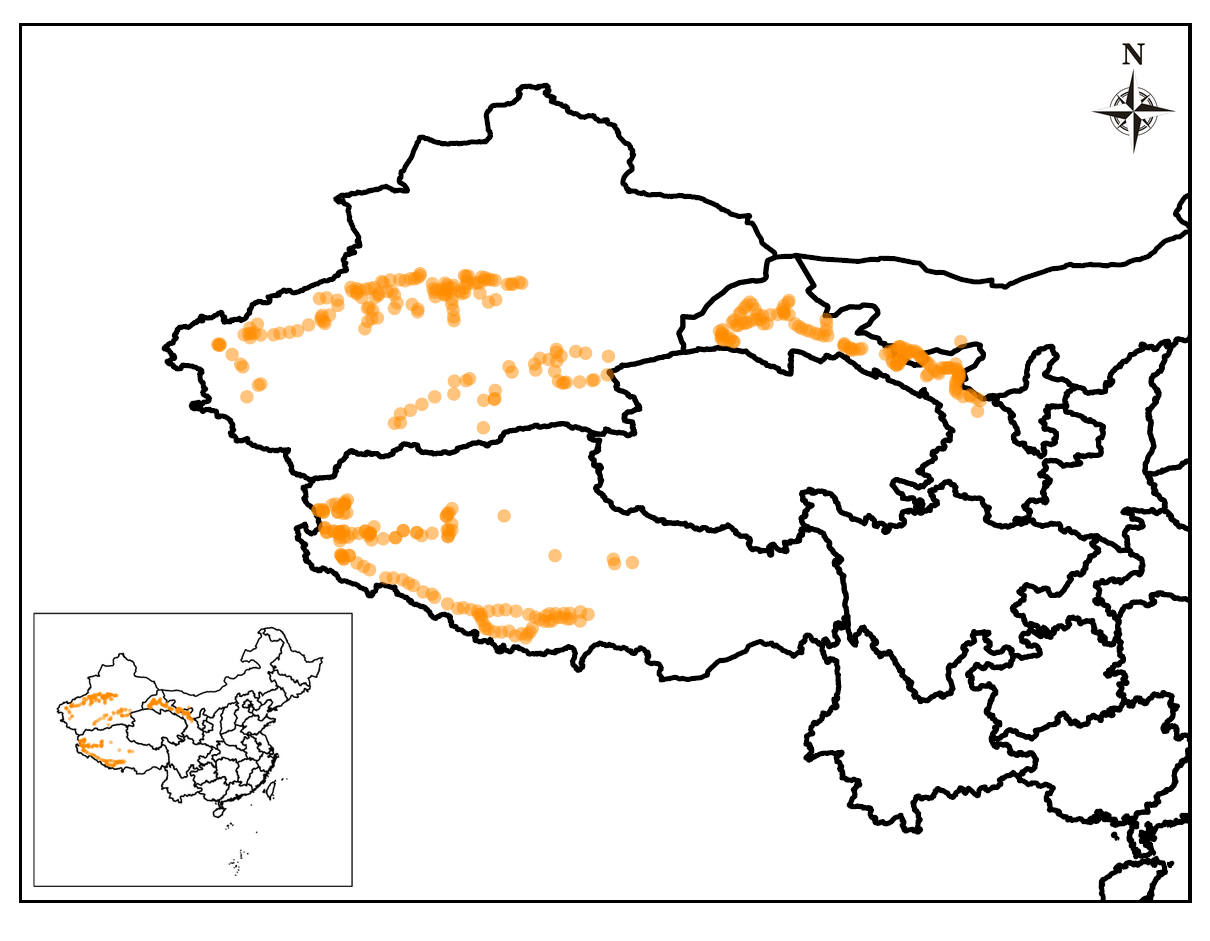


Figure S1 Study region and study site distribution


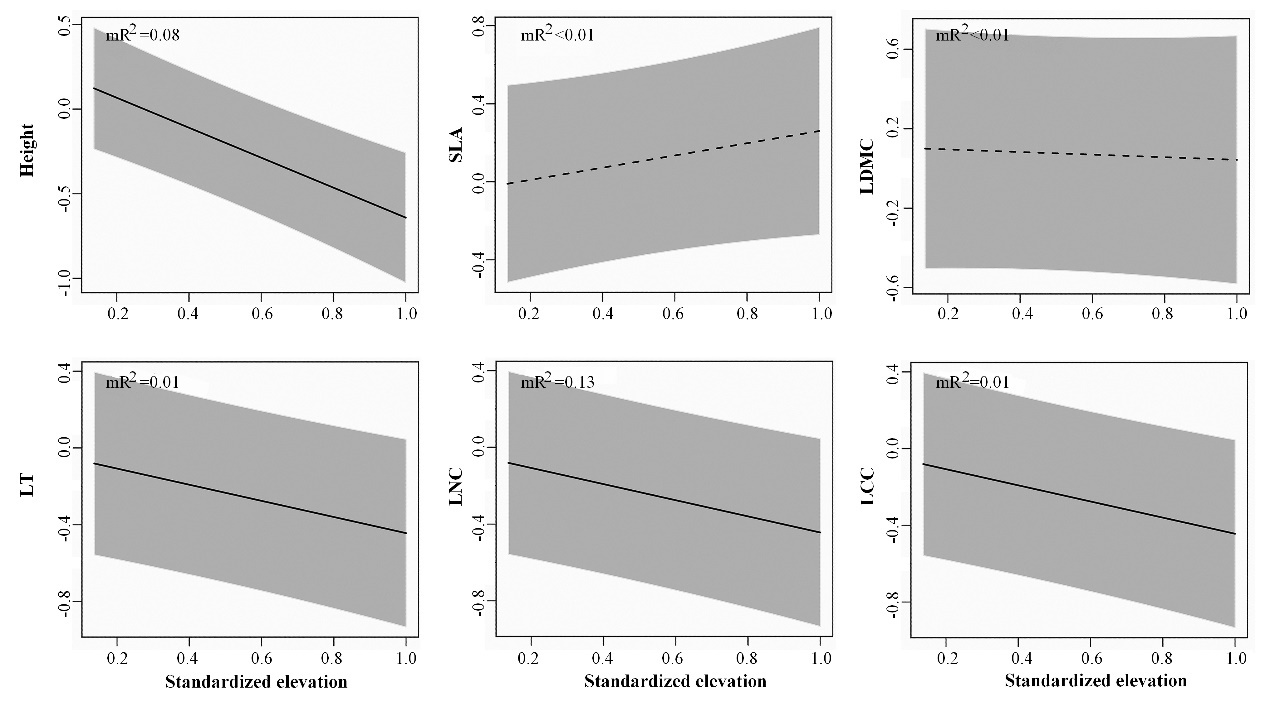


Figure S2 Trends in trait values for all species along the elevational gradients. Plant height, specific leaf area (SLA), leaf dry matter content (LDMC), leaf thickness (LT), leaf nitrogen content (LNC) and leaf carbon content (LCC) along elevational gradients (standardized value). The solid lines represent significant relationships and the dashed lines represent non-significant relationships. Gray areas are the 95% confidence intervals. mR^2^ represents Marginal R^2^ value. Trait values and elevation were standardized.


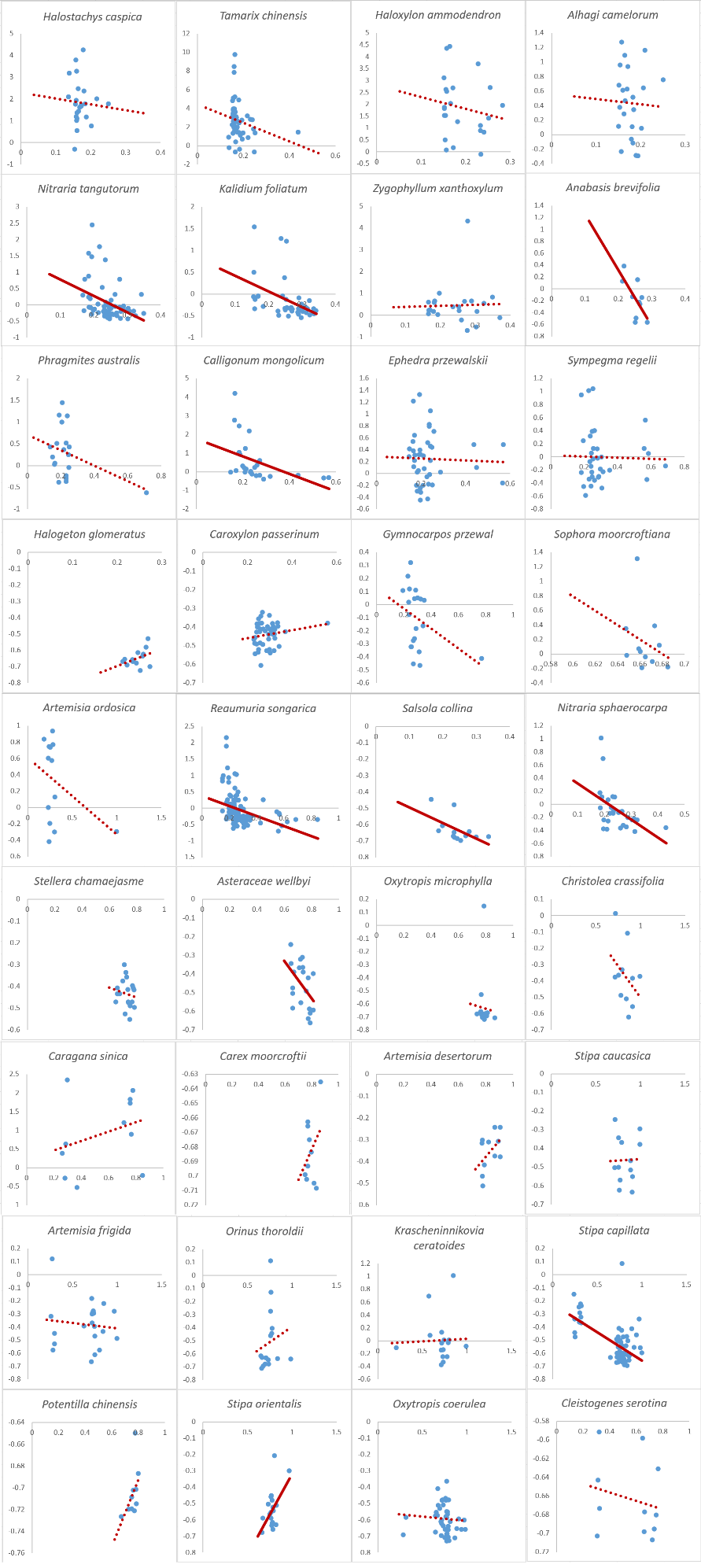


Figure S3 Plant height along elevation for individual species.


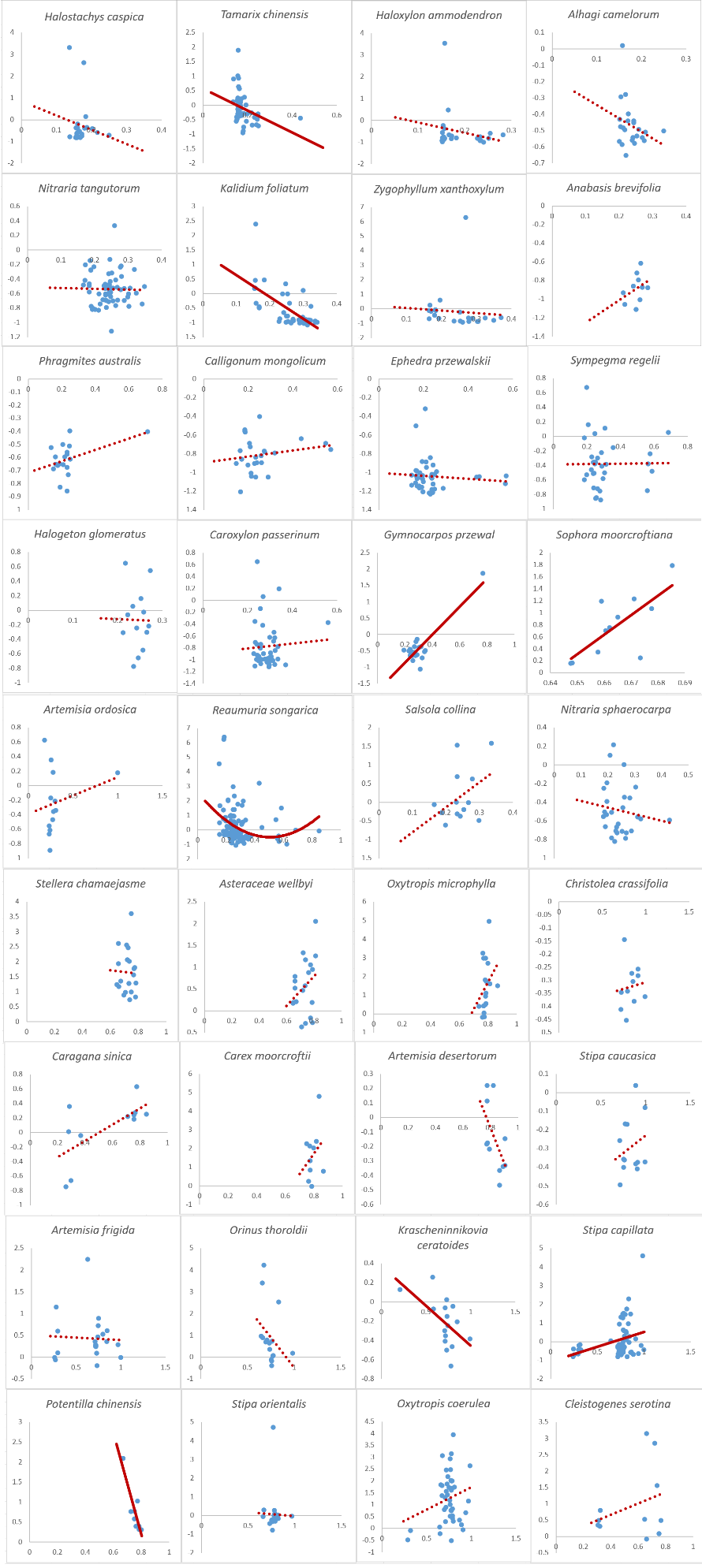


Figure S4 Specific leaf area (SLA) along elevation for individual species.


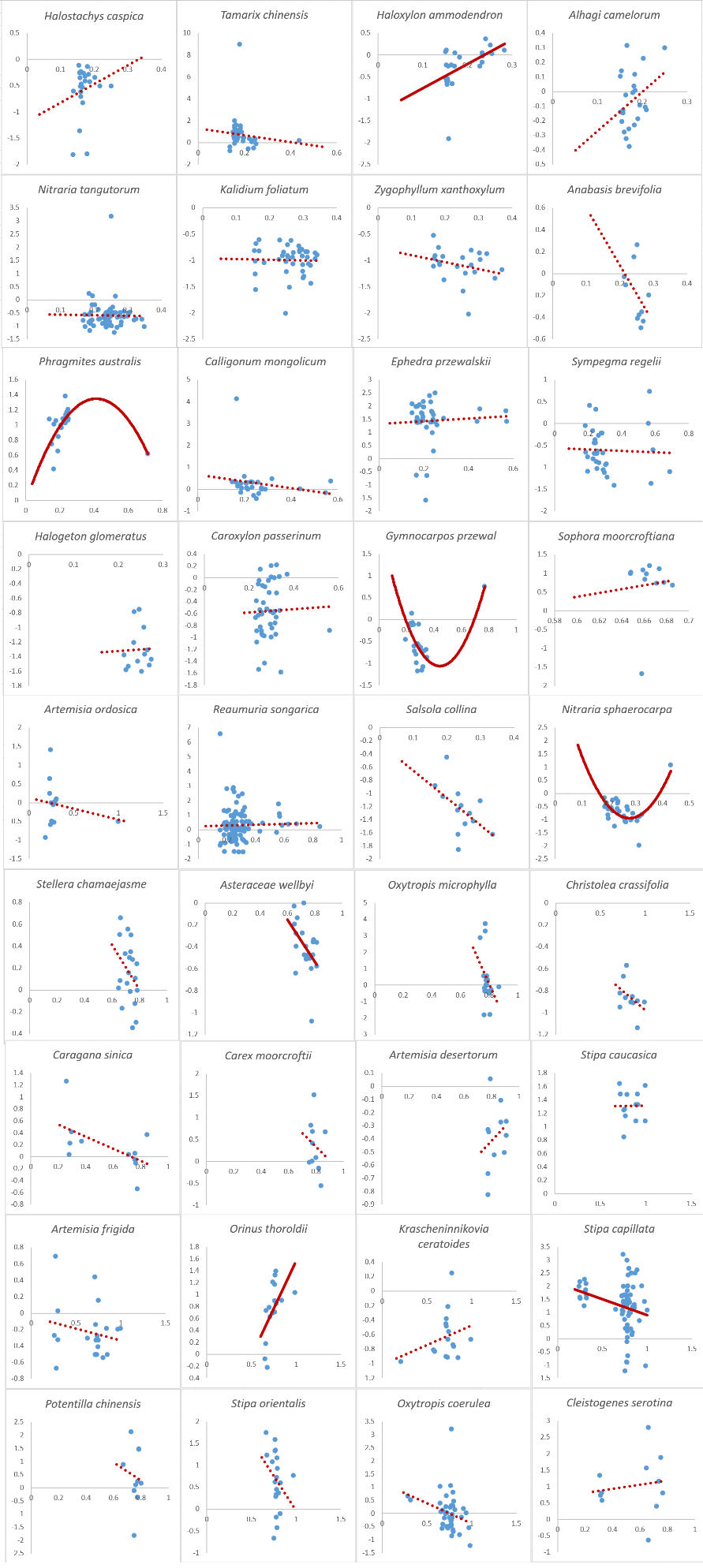


Figure S5 Leaf dry matter content (LDMC) along elevation for individual species.


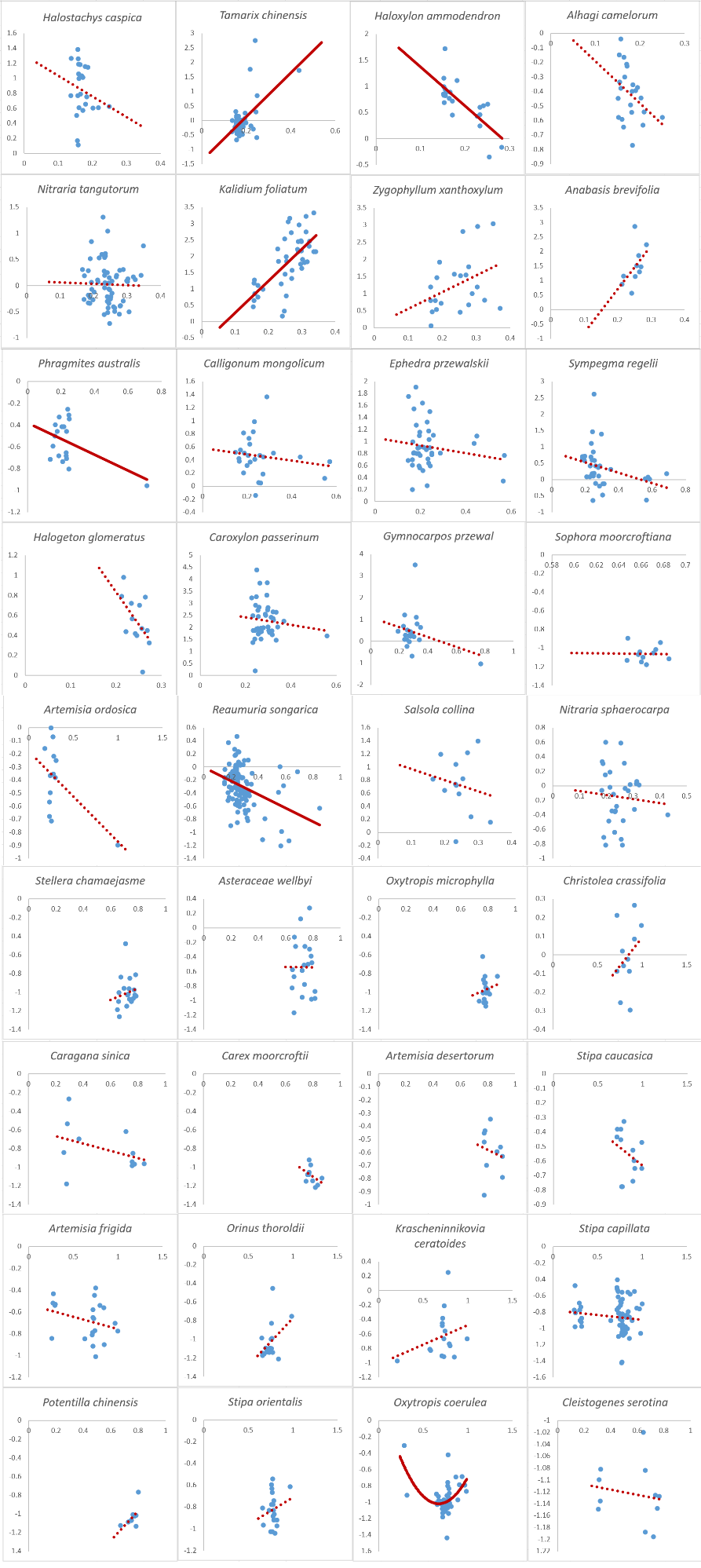


Figure S6 Leaf thickness (LT) along elevation for individual species.


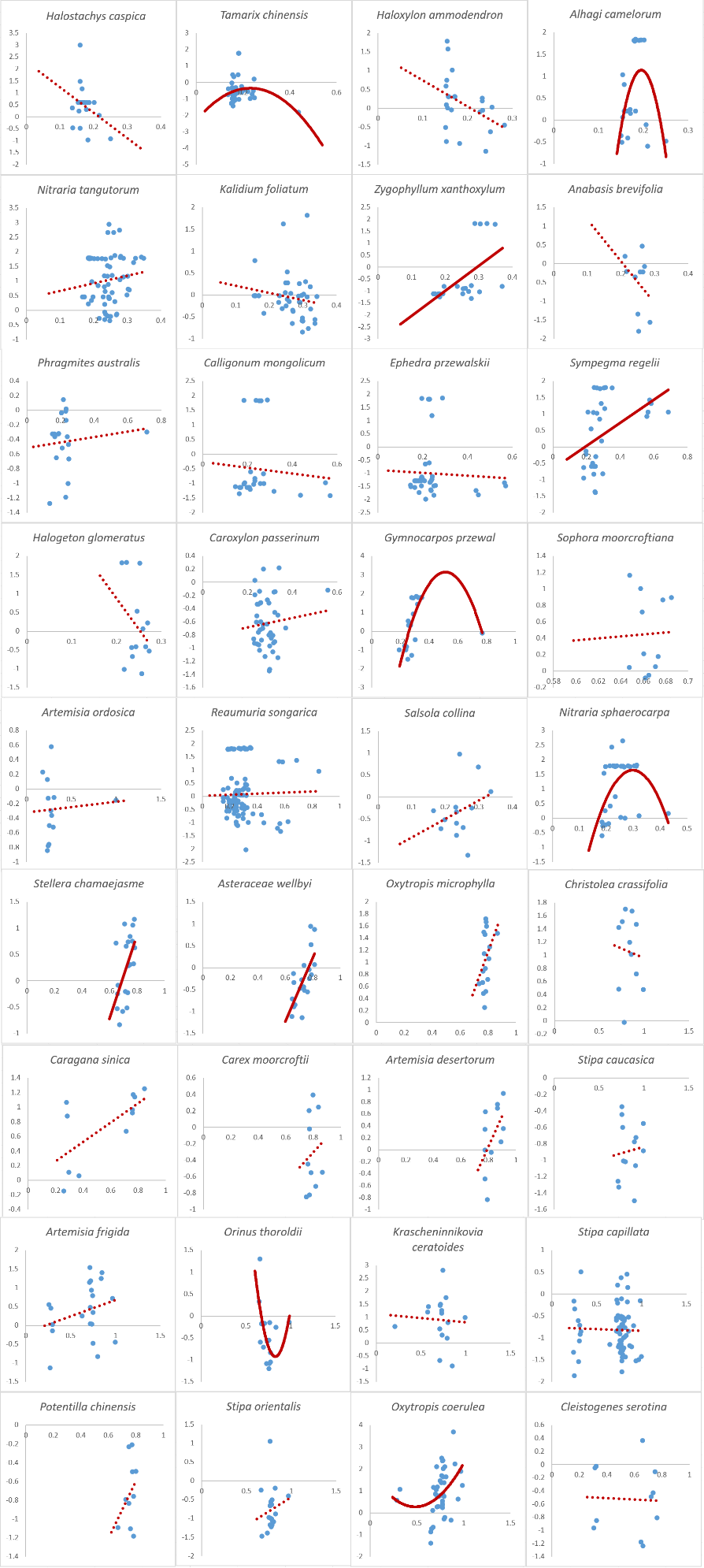


Figure S7 Leaf nitrogen content (LNC) along elevation for individual species.


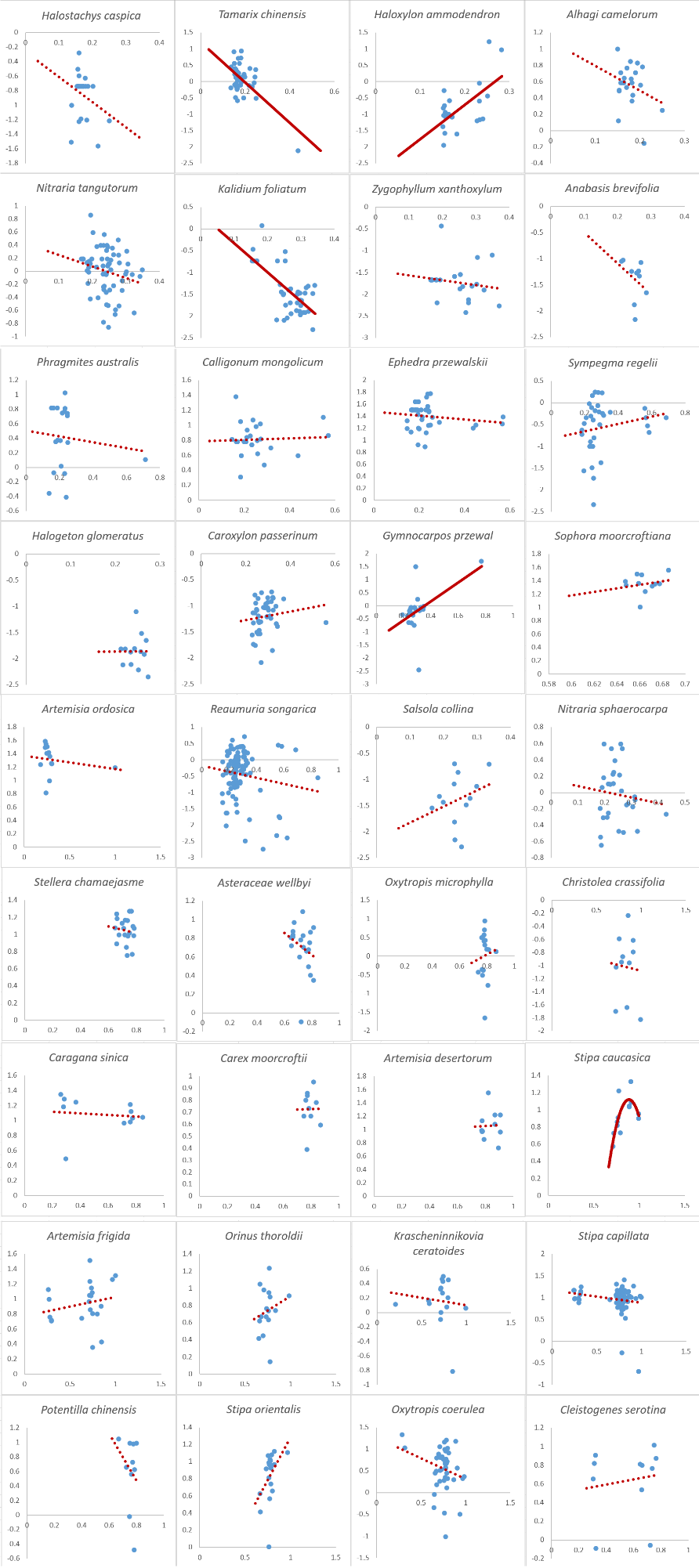


Figure S8 Leaf carbon content (LCC) along elevation for individual species.


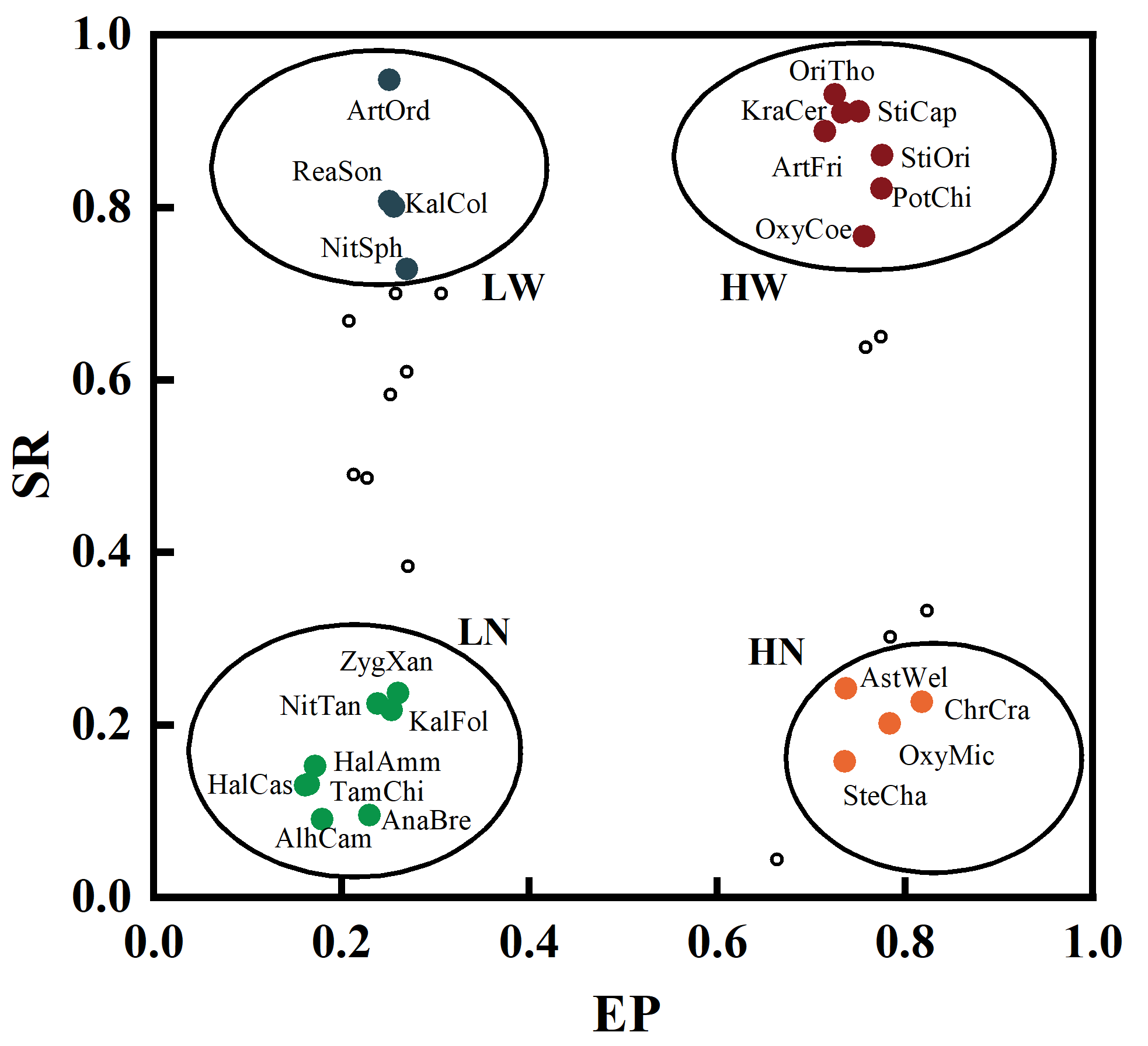


Figure S9 Desert plant species as characterized by their elevational preference (EP)and species range (SR).


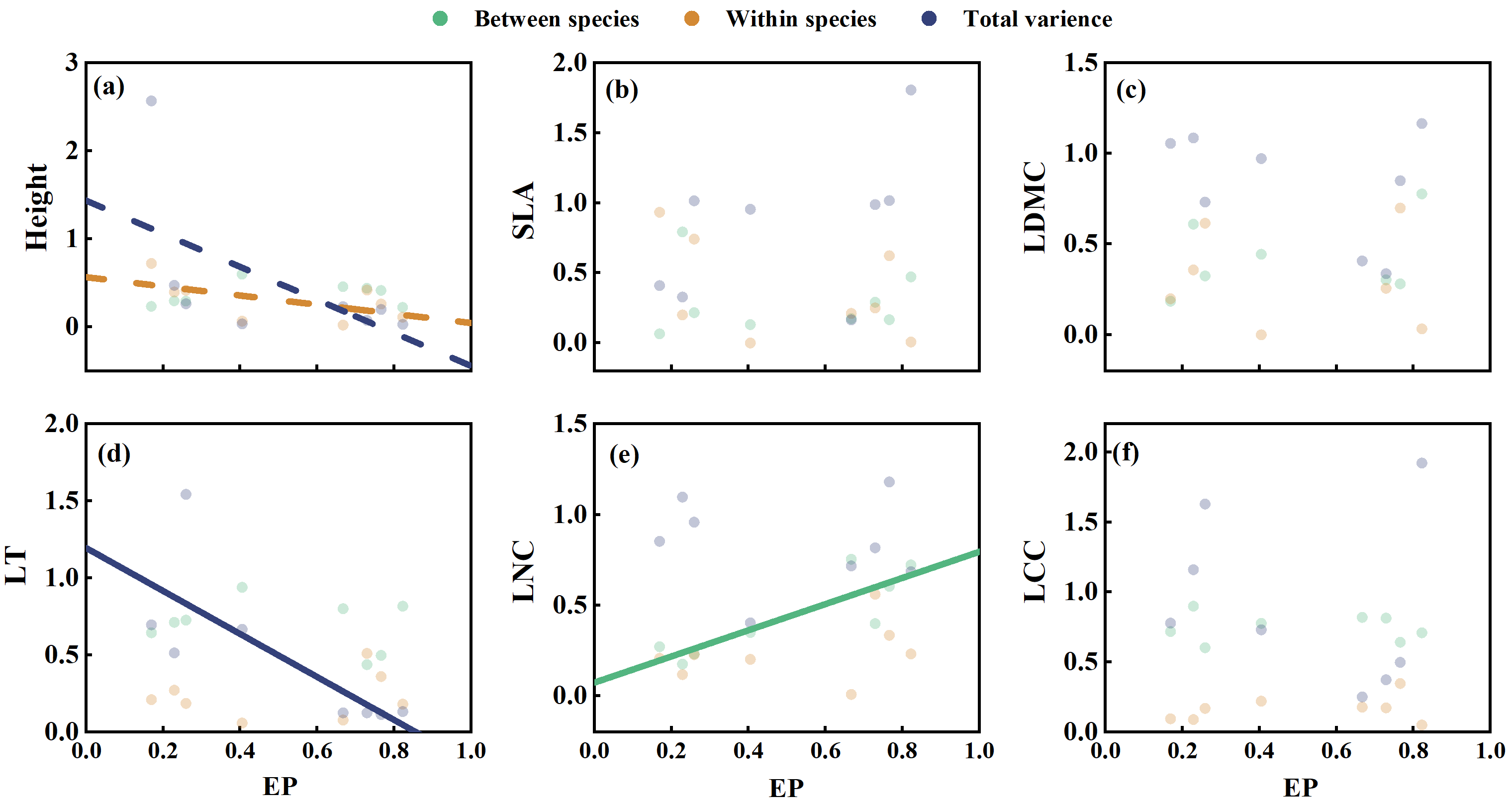


Figure S10 Relationship between the between species trait variation, within species trait variation and total trait variance of height, specific leaf area (SLA), leaf dry matter content (LDMC), leaf thickness (LT), leaf nitrogen content (LNC) and leaf carbon content (LCC) and species elevational preference (EP) gradients. Solid lines indicate significant trends (*p* <0.05), dashed lines indicate near significant trends (0.05 <*p* <0.10).


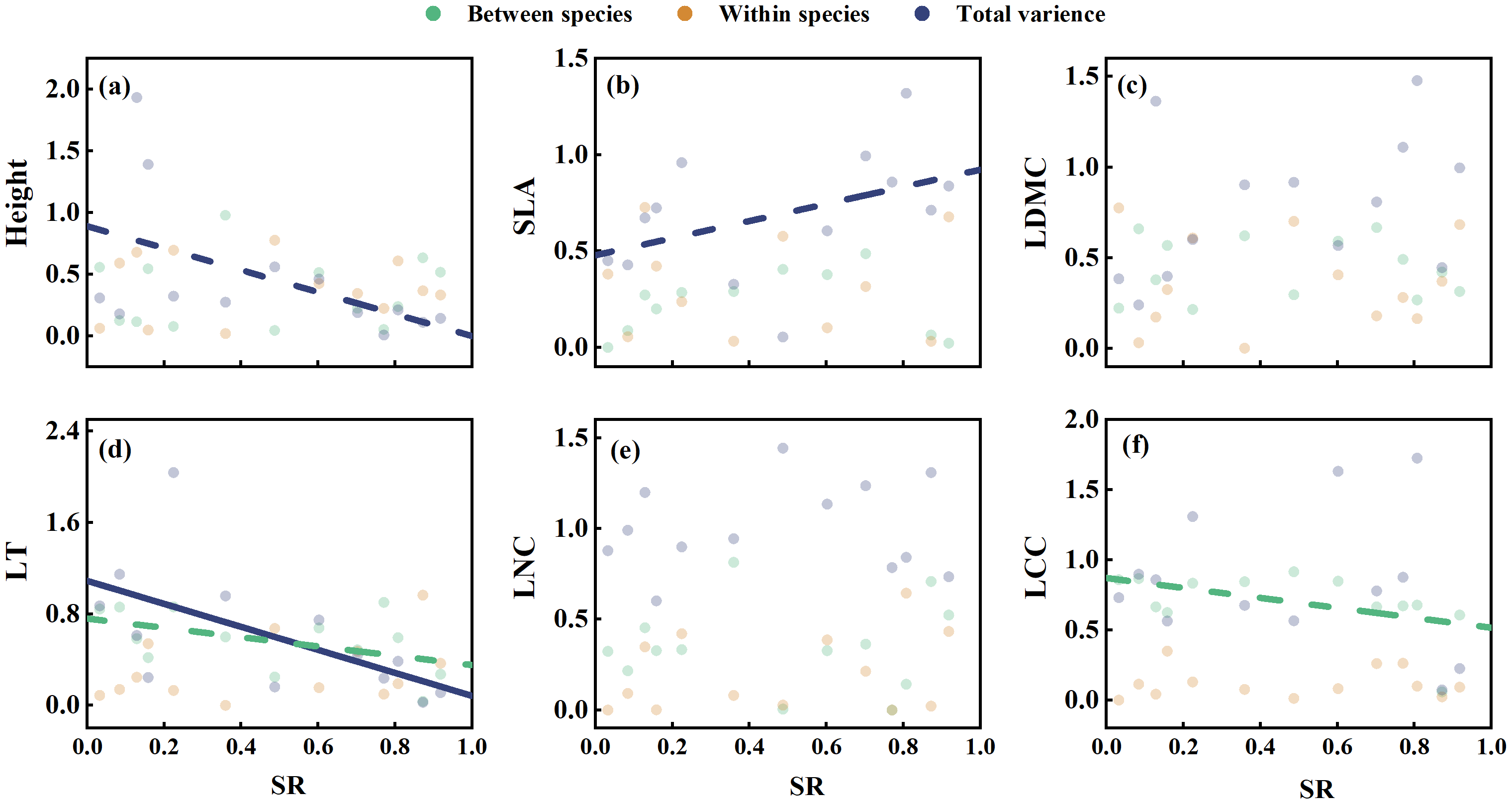


Figure S11 Relationship between the between species trait variation, within species trait variation and total trait variance of height, specific leaf area (SLA), leaf dry matter content (LDMC), leaf thickness (LT), leaf nitrogen content (LNC) and leaf carbon content (LCC) and species’ elevational range (SR) gradients. Solid lines indicate significant trends (*p* <0.05), dashed lines indicate near significant trends (0.05 <*p* <0.10).


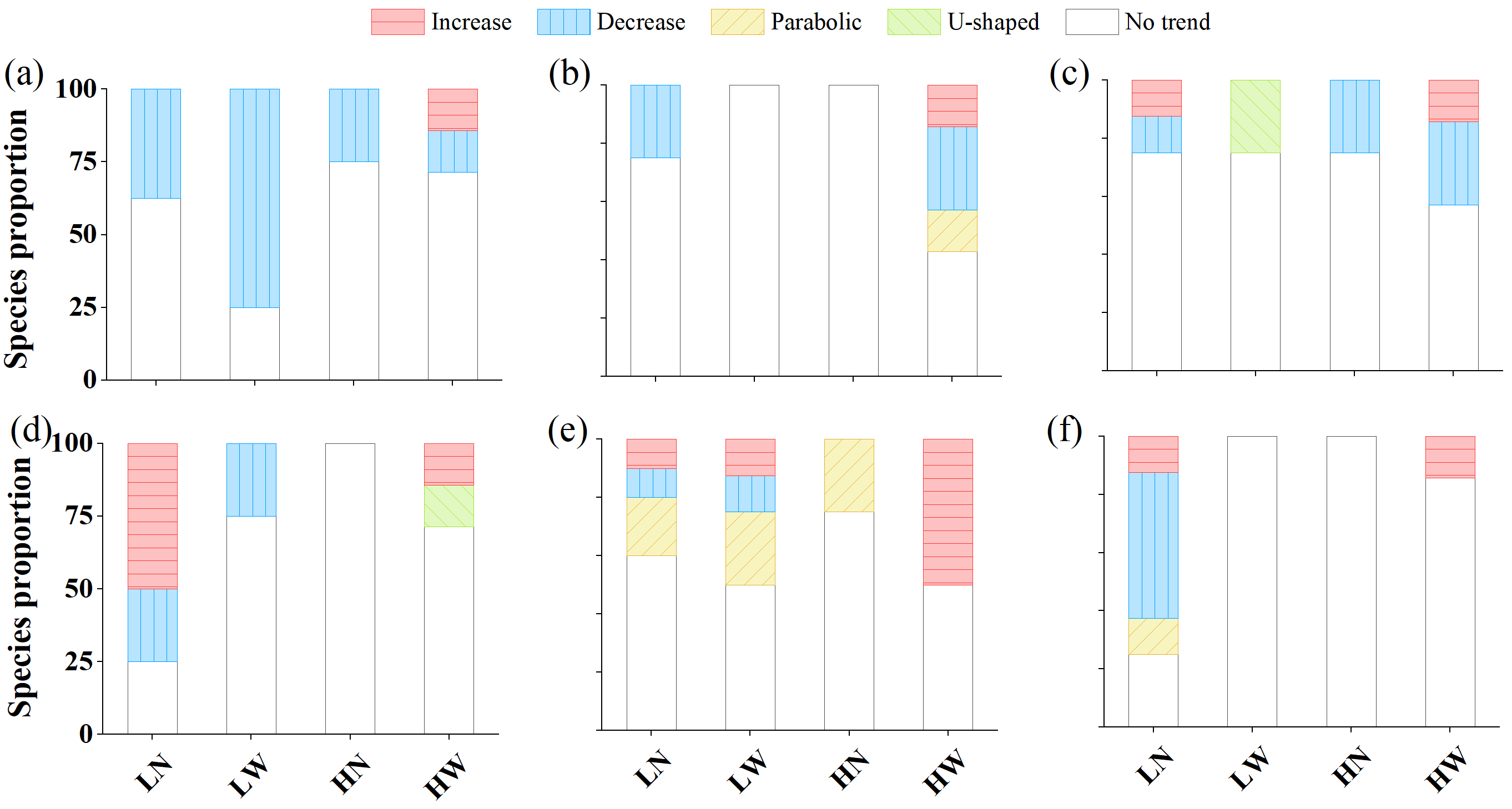


Figure S12 Summary of within-species variance patterns in functional traits: (a) plant height, (b) specific leaf area (SLA), (c) leaf dry matter content (LDMC), (d) leaf thickness (LT), (e) leaf nitrogen content (LNC) and (f) leaf carbon content (LCC).

Table S1 Sampling sites information

| **Site** | **longitude** | **Latitude** | **Elevation** | **Region** | **Year** |
| --- | --- | --- | --- | --- | --- |
| QH001 | 103.98 | 36.94 | 1995 | QH | 2019 |
| QH002 | 104.08 | 37.34 | 1590 | QH | 2019 |
| QH003 | 103.94 | 37.43 | 1776 | QH | 2019 |
| QH004 | 103.71 | 37.51 | 1929 | QH | 2019 |
| QH005 | 103.70 | 37.55 | 1783 | QH | 2019 |
| QH006 | 103.45 | 37.49 | 1887 | QH | 2019 |
| QH007 | 103.35 | 39.56 | 1269 | QH | 2019 |
| QH008 | 103.24 | 37.62 | 1817 | QH | 2019 |
| QH009 | 103.15 | 37.71 | 1677 | QH | 2019 |
| QH010 | 103.16 | 37.80 | 1654 | QH | 2019 |
| QH011 | 103.16 | 37.93 | 1614 | QH | 2019 |
| QH012 | 103.17 | 38.07 | 1512 | QH | 2019 |
| QH013 | 103.24 | 38.14 | 1488 | QH | 2019 |
| QH014 | 103.28 | 38.20 | 1469 | QH | 2019 |
| QH015 | 103.29 | 38.24 | 1461 | QH | 2019 |
| QH016 | 103.26 | 38.31 | 1448 | QH | 2019 |
| QH017 | 103.28 | 38.37 | 1490 | QH | 2019 |
| QH018 | 103.29 | 38.40 | 1435 | QH | 2019 |
| QH019 | 103.23 | 38.50 | 1387 | QH | 2019 |
| QH020 | 103.14 | 38.54 | 1385 | QH | 2019 |
| QH021 | 102.96 | 38.57 | 1384 | QH | 2019 |
| QH022 | 102.87 | 38.57 | 1384 | QH | 2019 |
| QH023 | 102.85 | 38.57 | 1386 | QH | 2019 |
| QH024 | 102.75 | 38.57 | 1381 | QH | 2019 |
| QH025 | 102.64 | 38.57 | 1375 | QH | 2019 |
| QH026 | 102.13 | 38.32 | 1755 | QH | 2019 |
| QH027 | 102.07 | 38.27 | 1917 | QH | 2019 |
| QH028 | 102.35 | 38.48 | 1472 | QH | 2019 |
| QH029 | 102.48 | 38.43 | 1513 | QH | 2019 |
| QH030 | 102.52 | 38.36 | 1542 | QH | 2019 |
| QH031 | 102.21 | 38.66 | 1488 | QH | 2019 |
| QH032 | 103.16 | 38.72 | 1355 | QH | 2019 |
| QH033 | 102.10 | 38.77 | 1529 | QH | 2019 |
| QH034 | 102.03 | 38.82 | 1482 | QH | 2019 |
| QH035 | 101.94 | 38.92 | 1400 | QH | 2019 |
| QH036 | 101.88 | 38.95 | 1379 | QH | 2019 |
| QH037 | 101.81 | 39.02 | 1390 | QH | 2019 |
| QH038 | 101.79 | 39.07 | 1442 | QH | 2019 |
| QH039 | 101.70 | 39.16 | 1540 | QH | 2019 |
| QH040 | 101.59 | 39.21 | 1430 | QH | 2019 |
| QH041 | 101.49 | 39.20 | 1433 | QH | 2019 |
| QH042 | 101.36 | 39.25 | 1433 | QH | 2019 |
| QH043 | 101.22 | 39.31 | 1462 | QH | 2019 |
| QH044 | 101.06 | 39.37 | 1513 | QH | 2019 |
| QH045 | 101.06 | 39.37 | 1512 | QH | 2019 |
| QH046 | 100.99 | 39.33 | 1564 | QH | 2019 |
| QH047 | 100.89 | 39.25 | 1703 | QH | 2019 |
| QH048 | 100.81 | 39.18 | 1893 | QH | 2019 |
| QH049 | 100.67 | 39.19 | 1827 | QH | 2019 |
| QH050 | 100.53 | 39.09 | 1517 | QH | 2019 |
| QH051 | 100.92 | 38.68 | 1861 | QH | 2019 |
| QH052 | 100.94 | 38.67 | 1910 | QH | 2019 |
| QH053 | 100.97 | 38.71 | 1821 | QH | 2019 |
| QH054 | 101.03 | 38.73 | 1855 | QH | 2019 |
| QH055 | 101.08 | 38.83 | 1877 | QH | 2019 |
| QH056 | 101.09 | 38.88 | 2197 | QH | 2019 |
| QH057 | 101.13 | 38.92 | 1990 | QH | 2019 |
| QH058 | 101.19 | 39.01 | 1793 | QH | 2019 |
| QH059 | 101.30 | 39.05 | 1647 | QH | 2019 |
| QH060 | 101.41 | 39.15 | 1475 | QH | 2019 |
| QH061 | 100.97 | 38.82 | 1716 | QH | 2019 |
| QH062 | 100.80 | 38.78 | 1666 | QH | 2019 |
| QH063 | 100.77 | 38.86 | 1607 | QH | 2019 |
| QH064 | 99.64 | 39.30 | 1446 | QH | 2019 |
| QH065 | 99.54 | 39.27 | 1599 | QH | 2019 |
| QH066 | 99.24 | 39.24 | 2029 | QH | 2019 |
| QH067 | 99.36 | 39.25 | 1718 | QH | 2019 |
| QH068 | 99.23 | 39.31 | 1768 | QH | 2019 |
| QH069 | 99.17 | 39.32 | 1707 | QH | 2019 |
| QH070 | 98.96 | 39.39 | 1654 | QH | 2019 |
| QH071 | 98.97 | 39.43 | 1600 | QH | 2019 |
| QH072 | 98.98 | 39.48 | 1536 | QH | 2019 |
| QH073 | 98.36 | 39.78 | 1573 | QH | 2019 |
| QH074 | 98.19 | 39.78 | 1752 | QH | 2019 |
| QH075 | 98.03 | 39.83 | 1798 | QH | 2019 |
| QH076 | 97.90 | 39.86 | 1912 | QH | 2019 |
| QH077 | 97.69 | 39.94 | 1774 | QH | 2019 |
| QH078 | 97.55 | 39.99 | 1727 | QH | 2019 |
| QH079 | 97.37 | 40.05 | 1597 | QH | 2019 |
| QH080 | 97.27 | 40.13 | 1602 | QH | 2019 |
| QH081 | 97.12 | 40.22 | 1524 | QH | 2019 |
| QH082 | 96.91 | 40.56 | 1397 | QH | 2019 |
| QH083 | 96.73 | 40.59 | 1390 | QH | 2019 |
| QH084 | 96.72 | 40.64 | 1439 | QH | 2019 |
| QH085 | 96.69 | 40.68 | 1506 | QH | 2019 |
| QH086 | 96.66 | 40.77 | 1546 | QH | 2019 |
| QH087 | 96.72 | 40.91 | 1711 | QH | 2019 |
| QH088 | 96.87 | 41.02 | 1851 | QH | 2019 |
| QH089 | 96.90 | 41.10 | 1929 | QH | 2019 |
| QH090 | 96.48 | 40.58 | 1336 | QH | 2019 |
| QH091 | 96.20 | 40.56 | 1261 | QH | 2019 |
| QH092 | 95.91 | 40.52 | 1199 | QH | 2019 |
| QH093 | 95.94 | 40.22 | 1383 | QH | 2019 |
| QH094 | 96.08 | 40.24 | 1312 | QH | 2019 |
| QH095 | 95.84 | 40.38 | 1270 | QH | 2019 |
| QH096 | 95.69 | 40.40 | 1154 | QH | 2019 |
| QH097 | 95.49 | 40.34 | 1129 | QH | 2019 |
| QH098 | 95.34 | 40.28 | 1108 | QH | 2019 |
| QH099 | 95.28 | 40.25 | 1121 | QH | 2019 |
| QH100 | 95.16 | 40.24 | 1122 | QH | 2019 |
| QH101 | 95.00 | 40.17 | 1096 | QH | 2019 |
| QH102 | 94.91 | 40.16 | 1095 | QH | 2019 |
| QH103 | 94.77 | 40.28 | 1084 | QH | 2019 |
| QH104 | 94.84 | 40.36 | 1064 | QH | 2019 |
| QH105 | 95.04 | 40.59 | 1076 | QH | 2019 |
| QH106 | 95.10 | 40.66 | 1135 | QH | 2019 |
| QH107 | 95.22 | 40.82 | 1370 | QH | 2019 |
| QH108 | 95.31 | 40.93 | 1559 | QH | 2019 |
| QH109 | 95.43 | 41.04 | 1713 | QH | 2019 |
| QH110 | 95.53 | 40.95 | 1583 | QH | 2019 |
| QH111 | 95.63 | 40.77 | 1343 | QH | 2019 |
| QH112 | 95.74 | 40.57 | 1172 | QH | 2019 |
| QH113 | 94.41 | 40.00 | 1320 | QH | 2019 |
| QH114 | 94.30 | 39.95 | 1418 | QH | 2019 |
| QH115 | 94.39 | 39.87 | 1495 | QH | 2019 |
| QH116 | 94.54 | 39.76 | 1674 | QH | 2019 |
| QH117 | 94.70 | 39.67 | 1860 | QH | 2019 |
| QH118 | 94.83 | 39.56 | 2073 | QH | 2019 |
| QH119 | 94.81 | 39.55 | 2101 | QH | 2019 |
| QH120 | 94.65 | 39.60 | 2031 | QH | 2019 |
| QH121 | 94.44 | 39.62 | 1808 | QH | 2019 |
| QH122 | 94.36 | 39.66 | 1660 | QH | 2019 |
| QH123 | 94.36 | 39.74 | 1545 | QH | 2019 |
| QH124 | 94.38 | 39.83 | 1518 | QH | 2019 |
| HXS01 | 98.31 | 39.98 | 1488 | QH | 2020 |
| HXS02 | 98.32 | 40.22 | 1260 | QH | 2020 |
| HXS03 | 98.30 | 40.38 | 1271 | QH | 2020 |
| QZ001 | 88.40 | 38.00 | 4397 | QZ | 2020 |
| QZ002 | 88.46 | 38.01 | 4228 | QZ | 2020 |
| QZ003 | 88.54 | 38.03 | 4222 | QZ | 2020 |
| QZ004 | 89.04 | 38.04 | 3738 | QZ | 2020 |
| QZ005 | 88.25 | 38.07 | 4074 | QZ | 2020 |
| QZ006 | 84.34 | 38.07 | 1240 | QZ | 2020 |
| QZ007 | 89.52 | 38.10 | 3417 | QZ | 2020 |
| QZ008 | 89.59 | 38.12 | 3474 | QZ | 2020 |
| QZ009 | 90.08 | 38.31 | 3357 | QZ | 2020 |
| QZ010 | 90.12 | 39.01 | 3320 | QZ | 2020 |
| QZ011 | 89.27 | 39.12 | 1351 | QZ | 2020 |
| QZ012 | 88.90 | 39.19 | 979 | QZ | 2020 |
| QZ013 | 88.35 | 39.02 | 873 | QZ | 2020 |
| QZ014 | 88.17 | 39.27 | 813 | QZ | 2020 |
| QZ015 | 88.17 | 39.16 | 821 | QZ | 2020 |
| QZ016 | 88.11 | 38.44 | 3358 | QZ | 2020 |
| QZ017 | 88.18 | 38.82 | 1186 | QZ | 2020 |
| QZ019 | 87.40 | 38.49 | 1291 | QZ | 2020 |
| QZ020 | 87.35 | 38.71 | 983 | QZ | 2020 |
| QZ021 | 86.50 | 38.42 | 1128 | QZ | 2020 |
| QZ022 | 86.39 | 38.62 | 1049 | QZ | 2020 |
| QZ023 | 85.43 | 36.32 | 5032 | QZ | 2020 |
| QZ024 | 85.44 | 37.35 | 2668 | QZ | 2020 |
| QZ025 | 85.84 | 37.39 | 3709 | QZ | 2020 |
| QZ026 | 85.87 | 37.72 | 1890 | QZ | 2020 |
| QZ027 | 85.86 | 37.42 | 3249 | QZ | 2020 |
| QZ028 | 87.56 | 38.93 | 877 | QZ | 2020 |
| QZ029 | 84.91 | 38.16 | 1225 | QZ | 2020 |
| QZ030 | 84.78 | 38.09 | 1243 | QZ | 2020 |
| QZ031 | 84.32 | 37.59 | 1485 | QZ | 2020 |
| QZ032 | 83.60 | 37.48 | 1374 | QZ | 2020 |
| QZ033 | 83.12 | 37.20 | 1424 | QZ | 2020 |
| QZ034 | 82.69 | 36.98 | 1531 | QZ | 2020 |
| QZ035 | 82.32 | 36.52 | 3382 | QZ | 2020 |
| QZ036 | 82.32 | 36.84 | 1622 | QZ | 2020 |
| QZ037 | 82.09 | 36.49 | 2597 | QZ | 2020 |
| QZ038 | 77.08 | 37.97 | 1390 | QZ | 2020 |
| QZ039 | 76.99 | 37.92 | 1566 | QZ | 2020 |
| QZ040 | 76.55 | 37.49 | 2553 | QZ | 2020 |
| QZ049 | 79.74 | 33.42 | 4253 | QZ | 2020 |
| QZ050 | 79.89 | 33.46 | 4280 | QZ | 2020 |
| QZ051 | 80.01 | 33.33 | 4997 | QZ | 2020 |
| QZ052 | 80.23 | 33.54 | 4289 | QZ | 2020 |
| QZ053 | 80.33 | 33.61 | 4540 | QZ | 2020 |
| QZ054 | 80.22 | 33.45 | 4491 | QZ | 2020 |
| QZ055 | 80.22 | 33.45 | 4491 | QZ | 2020 |
| QZ056 | 80.21 | 33.45 | 4707 | QZ | 2020 |
| QZ057 | 80.21 | 33.45 | 4707 | QZ | 2020 |
| QZ058 | 79.23 | 33.24 | 5894 | QZ | 2020 |
| QZ059 | 79.23 | 33.24 | 5894 | QZ | 2020 |
| QZ060 | 79.39 | 33.24 | 5748 | QZ | 2020 |
| QZ061 | 79.39 | 33.24 | 5748 | QZ | 2020 |
| QZ062 | 80.30 | 33.13 | 4412 | QZ | 2020 |
| QZ063 | 80.18 | 33.11 | 4370 | QZ | 2020 |
| QZ064 | 79.97 | 33.16 | 4341 | QZ | 2020 |
| QZ065 | 79.97 | 33.16 | 4341 | QZ | 2020 |
| QZ066 | 79.42 | 33.16 | 5930 | QZ | 2020 |
| QZ067 | 79.42 | 33.16 | 5930 | QZ | 2020 |
| QZ068 | 79.48 | 32.56 | 5413 | QZ | 2020 |
| QZ069 | 79.48 | 32.56 | 5413 | QZ | 2020 |
| QZ070 | 79.55 | 32.40 | 5295 | QZ | 2020 |
| QZ071 | 79.55 | 32.40 | 5295 | QZ | 2020 |
| QZ072 | 80.02 | 32.42 | 4263 | QZ | 2020 |
| QZ073 | 80.02 | 32.42 | 4263 | QZ | 2020 |
| QZ074 | 79.81 | 32.44 | 4230 | QZ | 2020 |
| QZ075 | 79.81 | 32.44 | 4230 | QZ | 2020 |
| QZ076 | 80.02 | 32.34 | 4626 | QZ | 2020 |
| QZ077 | 80.02 | 32.34 | 4626 | QZ | 2020 |
| QZ078 | 80.04 | 32.21 | 4253 | QZ | 2020 |
| QZ079 | 80.04 | 32.09 | 4270 | QZ | 2020 |
| QZ080 | 80.08 | 31.55 | 5385 | QZ | 2020 |
| QZ081 | 80.08 | 31.55 | 5385 | QZ | 2020 |
| QZ082 | 80.12 | 31.50 | 5147 | QZ | 2020 |
| QZ083 | 80.12 | 31.50 | 5147 | QZ | 2020 |
| QZ084 | 80.19 | 31.40 | 5080 | QZ | 2020 |
| QZ085 | 80.19 | 31.40 | 5080 | QZ | 2020 |
| QZ086 | 80.39 | 31.53 | 4594 | QZ | 2020 |
| QZ087 | 80.39 | 31.53 | 4594 | QZ | 2020 |
| QZ088 | 80.18 | 32.44 | 4355 | QZ | 2020 |
| QZ089 | 80.18 | 32.44 | 4355 | QZ | 2020 |
| QZ090 | 80.21 | 32.21 | 5272 | QZ | 2020 |
| QZ091 | 80.49 | 32.35 | 4663 | QZ | 2020 |
| QZ092 | 80.36 | 32.22 | 5569 | QZ | 2020 |
| QZ093 | 80.75 | 32.36 | 4539 | QZ | 2020 |
| QZ094 | 81.00 | 32.44 | 4497 | QZ | 2020 |
| QZ095 | 81.00 | 32.44 | 4497 | QZ | 2020 |
| QZ096 | 81.19 | 32.38 | 4556 | QZ | 2020 |
| QZ097 | 81.12 | 32.26 | 5023 | QZ | 2020 |
| QZ098 | 81.16 | 32.34 | 4523 | QZ | 2020 |
| QZ099 | 81.16 | 32.34 | 4523 | QZ | 2020 |
| QZ100 | 81.32 | 32.61 | 4872 | QZ | 2020 |
| QZ101 | 81.19 | 32.34 | 4531 | QZ | 2020 |
| QZ102 | 81.23 | 32.11 | 4927 | QZ | 2020 |
| QZ103 | 81.67 | 32.16 | 4882 | QZ | 2020 |
| QZ104 | 82.13 | 32.20 | 4698 | QZ | 2020 |
| QZ105 | 82.13 | 32.20 | 4698 | QZ | 2020 |
| QZ106 | 82.42 | 32.46 | 4605 | QZ | 2020 |
| QZ107 | 82.42 | 32.46 | 4605 | QZ | 2020 |
| QZ108 | 82.93 | 32.39 | 4463 | QZ | 2020 |
| QZ109 | 82.93 | 32.39 | 4463 | QZ | 2020 |
| QZ110 | 83.11 | 32.27 | 5844 | QZ | 2020 |
| QZ111 | 83.50 | 32.36 | 4503 | QZ | 2020 |
| QZ112 | 84.10 | 32.24 | 4600 | QZ | 2020 |
| QZ113 | 84.10 | 32.24 | 4600 | QZ | 2020 |
| QZ114 | 84.25 | 32.48 | 4573 | QZ | 2020 |
| QZ115 | 84.15 | 32.34 | 4452 | QZ | 2020 |
| QZ116 | 84.24 | 32.70 | 4620 | QZ | 2020 |
| QZ117 | 84.12 | 32.48 | 4753 | QZ | 2020 |
| QZ118 | 84.05 | 33.00 | 4621 | QZ | 2020 |
| QZ119 | 84.05 | 33.00 | 4621 | QZ | 2020 |
| QZ120 | 84.25 | 33.29 | 4626 | QZ | 2020 |
| QZ121 | 84.09 | 33.12 | 4690 | QZ | 2020 |
| QZ122 | 84.14 | 33.11 | 4642 | QZ | 2020 |
| QZ123 | 84.07 | 32.49 | 4756 | QZ | 2020 |
| QZ124 | 86.21 | 33.01 | 4873 | QZ | 2020 |
| QZ125 | 88.12 | 31.52 | 5145 | QZ | 2020 |
| QZ126 | 90.31 | 31.39 | 4631 | QZ | 2020 |
| QZ127 | 90.36 | 31.22 | 4665 | QZ | 2020 |
| QZ128 | 91.02 | 31.26 | 4769 | QZ | 2020 |
| TSP001 | 86.54 | 41.71 | 1162 | TSP | 2021 |
| TSP002 | 86.77 | 41.79 | 1082 | TSP | 2021 |
| TSP003 | 86.87 | 41.75 | 1092 | TSP | 2021 |
| TSP004 | 86.47 | 41.73 | 1113 | TSP | 2021 |
| TSP005 | 86.34 | 41.71 | 1167 | TSP | 2021 |
| TSP006 | 85.89 | 41.13 | 895 | TSP | 2021 |
| TSP007 | 85.62 | 41.04 | 902 | TSP | 2021 |
| TSP008 | 85.87 | 41.86 | 1013 | TSP | 2021 |
| TSP009 | 85.73 | 41.88 | 994 | TSP | 2021 |
| TSP010 | 85.62 | 41.90 | 984 | TSP | 2021 |
| TSP011 | 85.45 | 41.86 | 912 | TSP | 2021 |
| TSP012 | 85.19 | 41.78 | 904 | TSP | 2021 |
| TSP013 | 85.30 | 41.38 | 906 | TSP | 2021 |
| TSP014 | 85.28 | 41.75 | 899 | TSP | 2021 |
| TSP015 | 85.49 | 41.96 | 1086 | TSP | 2021 |
| TSP016 | 85.36 | 41.98 | 984 | TSP | 2021 |
| TSP017 | 85.08 | 41.74 | 906 | TSP | 2021 |
| TSP018 | 84.73 | 41.59 | 920 | TSP | 2021 |
| TSP019 | 84.73 | 41.40 | 916 | TSP | 2021 |
| TSP020 | 84.62 | 41.36 | 922 | TSP | 2021 |
| TSP021 | 84.53 | 41.45 | 922 | TSP | 2021 |
| TSP022 | 84.72 | 42.03 | 1118 | TSP | 2021 |
| TSP023 | 84.79 | 42.01 | 1022 | TSP | 2021 |
| TSP024 | 84.87 | 42.02 | 1071 | TSP | 2021 |
| TSP025 | 84.80 | 41.84 | 929 | TSP | 2021 |
| TSP026 | 84.82 | 41.73 | 917 | TSP | 2021 |
| TSP027 | 84.28 | 41.57 | 934 | TSP | 2021 |
| TSP028 | 84.32 | 41.67 | 952 | TSP | 2021 |
| TSP029 | 83.99 | 41.75 | 976 | TSP | 2021 |
| TSP030 | 84.07 | 41.69 | 955 | TSP | 2021 |
| TSP031 | 84.07 | 41.58 | 940 | TSP | 2021 |
| TSP032 | 84.02 | 41.50 | 938 | TSP | 2021 |
| TSP033 | 83.94 | 41.33 | 936 | TSP | 2021 |
| TSP034 | 84.00 | 41.31 | 933 | TSP | 2021 |
| TSP035 | 84.22 | 41.42 | 929 | TSP | 2021 |
| TSP036 | 84.25 | 41.14 | 933 | TSP | 2021 |
| TSP038 | 84.33 | 40.34 | 933 | TSP | 2021 |
| TSP039 | 84.32 | 40.48 | 934 | TSP | 2021 |
| TSP040 | 84.30 | 40.75 | 933 | TSP | 2021 |
| TSP041 | 84.05 | 41.38 | 934 | TSP | 2021 |
| TSP042 | 83.88 | 41.52 | 939 | TSP | 2021 |
| TSP043 | 83.62 | 41.24 | 949 | TSP | 2021 |
| TSP044 | 83.58 | 41.41 | 946 | TSP | 2021 |
| TSP045 | 83.67 | 41.50 | 948 | TSP | 2021 |
| TSP046 | 83.55 | 41.54 | 954 | TSP | 2021 |
| TSP047 | 83.52 | 41.73 | 972 | TSP | 2021 |
| TSP048 | 82.84 | 41.92 | 1411 | TSP | 2021 |
| TSP049 | 82.96 | 41.95 | 1485 | TSP | 2021 |
| TSP050 | 83.05 | 42.03 | 1446 | TSP | 2021 |
| TSP051 | 83.05 | 42.11 | 1502 | TSP | 2021 |
| TSP052 | 82.70 | 41.92 | 1481 | TSP | 2021 |
| TSP053 | 82.49 | 41.84 | 1244 | TSP | 2021 |
| TSP054 | 82.28 | 41.88 | 1384 | TSP | 2021 |
| TSP055 | 81.94 | 41.88 | 1416 | TSP | 2021 |
| TSP056 | 81.62 | 41.81 | 1444 | TSP | 2021 |
| TSP057 | 81.68 | 41.76 | 1350 | TSP | 2021 |
| TSP058 | 81.34 | 41.58 | 1501 | TSP | 2021 |
| TSP059 | 81.19 | 41.55 | 1438 | TSP | 2021 |
| TSP060 | 81.02 | 41.53 | 1401 | TSP | 2021 |
| TSP061 | 81.99 | 41.45 | 976 | TSP | 2021 |
| TSP062 | 82.09 | 41.32 | 980 | TSP | 2021 |
| TSP063 | 81.82 | 41.44 | 993 | TSP | 2021 |
| TSP064 | 81.37 | 41.22 | 1017 | TSP | 2021 |
| TSP065 | 81.49 | 41.30 | 1001 | TSP | 2021 |
| TSP066 | 81.50 | 41.37 | 1001 | TSP | 2021 |
| TSP067 | 82.96 | 40.68 | 961 | TSP | 2021 |
| TSP068 | 82.97 | 40.77 | 963 | TSP | 2021 |
| TSP069 | 82.75 | 40.95 | 969 | TSP | 2021 |
| TSP070 | 82.19 | 41.02 | 981 | TSP | 2021 |
| TSP071 | 82.09 | 40.80 | 986 | TSP | 2021 |
| TSP072 | 81.45 | 40.45 | 1010 | TSP | 2021 |
| TSP073 | 81.25 | 40.42 | 1016 | TSP | 2021 |
| TSP074 | 81.09 | 40.32 | 1031 | TSP | 2021 |
| TSP075 | 80.98 | 40.04 | 1053 | TSP | 2021 |
| TSP076 | 81.28 | 40.74 | 1014 | TSP | 2021 |
| TSP077 | 81.08 | 40.81 | 1027 | TSP | 2021 |
| TSP078 | 81.14 | 40.84 | 1027 | TSP | 2021 |
| TSP079 | 80.87 | 41.21 | 1112 | TSP | 2021 |
| TSP080 | 80.68 | 41.36 | 1221 | TSP | 2021 |
| TSP081 | 80.78 | 41.49 | 1366 | TSP | 2021 |
| TSP082 | 80.68 | 41.40 | 1258 | TSP | 2021 |
| TSP083 | 80.66 | 41.55 | 1442 | TSP | 2021 |
| TSP084 | 80.46 | 41.59 | 1698 | TSP | 2021 |
| TSP085 | 80.50 | 41.46 | 1409 | TSP | 2021 |
| TSP086 | 79.28 | 41.17 | 1475 | TSP | 2021 |
| TSP087 | 79.55 | 41.20 | 1364 | TSP | 2021 |
| TSP088 | 79.96 | 41.11 | 1183 | TSP | 2021 |
| TSP089 | 77.56 | 39.81 | 1159 | TSP | 2021 |
| TSP090 | 79.97 | 40.92 | 1145 | TSP | 2021 |
| TSP091 | 77.80 | 39.87 | 1152 | TSP | 2021 |
| TSP092 | 79.66 | 40.56 | 1061 | TSP | 2021 |
| TSP093 | 79.38 | 40.54 | 1099 | TSP | 2021 |
| TSP094 | 79.34 | 40.46 | 1090 | TSP | 2021 |
| TSP095 | 79.34 | 40.36 | 1071 | TSP | 2021 |
| TSP096 | 79.45 | 40.32 | 1069 | TSP | 2021 |
| TSP097 | 79.48 | 40.24 | 1070 | TSP | 2021 |
| TSP098 | 78.16 | 39.91 | 1171 | TSP | 2021 |
| TSP099 | 78.45 | 39.97 | 1154 | TSP | 2021 |
| TSP100 | 77.07 | 39.80 | 1180 | TSP | 2021 |
| TSP101 | 76.84 | 39.87 | 1253 | TSP | 2021 |
| TSP102 | 76.71 | 39.86 | 1241 | TSP | 2021 |
| TSP103 | 76.67 | 39.71 | 1201 | TSP | 2021 |
| TSP104 | 76.45 | 39.81 | 1261 | TSP | 2021 |
| TSP105 | 76.62 | 39.86 | 1289 | TSP | 2021 |
| TSP106 | 76.67 | 40.07 | 1762 | TSP | 2021 |
| TSP107 | 76.95 | 40.22 | 1651 | TSP | 2021 |
| TSP108 | 76.00 | 39.07 | 1359 | TSP | 2021 |
| TSP109 | 76.32 | 38.71 | 1449 | TSP | 2021 |
| TSP110 | 76.40 | 38.61 | 1452 | TSP | 2021 |
| TSP111 | 75.55 | 39.42 | 1527 | TSP | 2021 |
| TSP112 | 75.50 | 39.43 | 1560 | TSP | 2021 |
| TSP113 | 75.46 | 39.45 | 1602 | TSP | 2021 |
| TSP114 | 75.53 | 39.47 | 1574 | TSP | 2021 |
| TSP115 | 78.87 | 40.18 | 1096 | TSP | 2021 |
| YJ01 | 89.06 | 29.07 | 3980 | YJ | 2022 |
| YJ02 | 89.36 | 29.32 | 3933 | YJ | 2022 |
| YJ03 | 89.07 | 29.41 | 3844 | YJ | 2022 |
| YJ04 | 88.70 | 29.17 | 3909 | YJ | 2022 |
| YJ05 | 88.59 | 29.14 | 4019 | YJ | 2022 |
| YJ06 | 88.39 | 29.20 | 3901 | YJ | 2022 |
| YJ07 | 88.21 | 29.21 | 4000 | YJ | 2022 |
| YJ08 | 88.02 | 29.09 | 4246 | YJ | 2022 |
| YJ09 | 87.90 | 29.05 | 4064 | YJ | 2022 |
| YJ10 | 87.72 | 29.06 | 3980 | YJ | 2022 |
| YJ11 | 87.55 | 29.13 | 3994 | YJ | 2022 |
| YJ12 | 87.27 | 28.81 | 4597 | YJ | 2022 |
| YJ13 | 87.19 | 28.66 | 4354 | YJ | 2022 |
| YJ14 | 86.63 | 28.51 | 4386 | YJ | 2022 |
| YJ15 | 87.03 | 28.44 | 4135 | YJ | 2022 |
| YJ16 | 87.08 | 28.57 | 4207 | YJ | 2022 |
| YJ17 | 86.87 | 28.59 | 4278 | YJ | 2022 |
| YJ18 | 86.39 | 28.69 | 4372 | YJ | 2022 |
| YJ19 | 86.11 | 28.65 | 4586 | YJ | 2022 |
| YJ20 | 85.85 | 28.66 | 4650 | YJ | 2022 |
| YJ21 | 85.62 | 28.76 | 4608 | YJ | 2022 |
| YJ22 | 85.51 | 28.79 | 4564 | YJ | 2022 |
| YJ23 | 85.48 | 28.91 | 4823 | YJ | 2022 |
| YJ24 | 85.38 | 29.10 | 4648 | YJ | 2022 |
| YJ25 | 85.35 | 29.23 | 4543 | YJ | 2022 |
| YJ26 | 85.25 | 29.31 | 4455 | YJ | 2022 |
| YJ27 | 85.20 | 29.44 | 4652 | YJ | 2022 |
| YJ28 | 84.96 | 29.50 | 4522 | YJ | 2022 |
| YJ29 | 84.68 | 29.57 | 4594 | YJ | 2022 |
| YJ30 | 84.48 | 29.54 | 4518 | YJ | 2022 |
| YJ31 | 84.09 | 29.71 | 4542 | YJ | 2022 |
| YJ32 | 83.60 | 29.96 | 4601 | YJ | 2022 |
| YJ33 | 83.50 | 30.08 | 4592 | YJ | 2022 |
| YJ34 | 83.17 | 30.16 | 4583 | YJ | 2022 |
| YJ35 | 82.81 | 30.40 | 4679 | YJ | 2022 |
| YJ36 | 82.62 | 30.49 | 4821 | YJ | 2022 |
| YJ37 | 82.40 | 30.62 | 5080 | YJ | 2022 |
| YJ38 | 82.05 | 30.69 | 4787 | YJ | 2022 |
| YJ39 | 81.77 | 30.69 | 4740 | YJ | 2022 |
| YJ40 | 81.16 | 30.99 | 4686 | YJ | 2022 |
| YJ41 | 80.93 | 31.12 | 4658 | YJ | 2022 |
| YJ42 | 80.69 | 31.25 | 4531 | YJ | 2022 |
| YJ43 | 85.34 | 29.33 | 4449 | YJ | 2022 |
| YJ44 | 85.64 | 29.44 | 4966 | YJ | 2022 |
| YJ45 | 85.98 | 29.47 | 4849 | YJ | 2022 |
| YJ46 | 86.27 | 29.49 | 4733 | YJ | 2022 |
| YJ47 | 86.66 | 29.44 | 4570 | YJ | 2022 |
| YJ48 | 87.14 | 29.31 | 4313 | YJ | 2022 |
| YJ49 | 87.38 | 29.21 | 4267 | YJ | 2022 |
| YJ50 | 87.78 | 29.27 | 3944 | YJ | 2022 |
| YJ51 | 87.91 | 29.34 | 3918 | YJ | 2022 |
| YJ52 | 88.14 | 29.34 | 3926 | YJ | 2022 |
| YJ53 | 88.46 | 29.35 | 3857 | YJ | 2022 |
| YJ54 | 88.68 | 29.36 | 3841 | YJ | 2022 |

Table S2 Desert plant species as from Figure 3 for the different groups of habitat preference

| **Species name** | **species code** | **functional group** | **EP** | **SR** | **GROUP** |
| --- | --- | --- | --- | --- | --- |
| *Artemisia ordosica* | ArtOrd | shrub | low | wide | LW |
| *Kali collinum* | KalCol | forb | low | wide | LW |
| *Nitraria sphaerocarpa* | NitSph | shrub | low | wide | LW |
| *Reaumuria songarica* | ReaSon | shrub | low | wide | LW |
| *Alhagi camelorum* | AlhCam | shrub | low | narrow | LN |
| *Anabasis brevifolia* | AnaBre | shrub | low | narrow | LN |
| *Halostachys caspica* | HalCas | shrub | low | narrow | LN |
| *Haloxylon ammodendron* | HalAmm | shrub | low | narrow | LN |
| *Kalidium foliatum* | KalFol | shrub | low | narrow | LN |
| *Nitraria tangutorum* | NitTan | shrub | low | narrow | LN |
| *Tamarix chinensis* | TamChi | shrub | low | narrow | LN |
| *Zygophyllum xanthoxylum* | ZygXan | shrub | low | narrow | LN |
| *Artemisia frigida* | ArtFri | forb | high | wide | HW |
| *Krascheninnikovia ceratoides* | KraCer | shrub | high | wide | HW |
| *Orinus thoroldii* | OriTho | graminoid | high | wide | HW |
| *Oxytropis coerulea* | OxyCoe | forb | high | wide | HW |
| *Potentilla chinensis* | PotChi | forb | high | wide | HW |
| *Stipa capillata* | StiCap | graminoid | high | wide | HW |
| *Stipa orientalis* | StiOri | graminoid | high | wide | HW |
| *Asteraceae wellbyi* | AstWel | shrub | high | narrow | HN |
| *Christolea crassifolia* | ChrCra | forb | high | narrow | HN |
| *Oxytropis microphylla* | OxyMic | forb | high | narrow | HN |
| *Stellera chamaejasme* | SteCha | forb | high | narrow | HN |

Table S3 RDA analysis results of cliamte factors and soil characteristics

| Explanatory variable | LN | | LW | | HN | | HW | |
| --- | --- | --- | --- | --- | --- | --- | --- | --- |
|  | R^2^ | *P* | R^2^ | *P* | R^2^ | *P* | R^2^ | *P* |
| Elevation | 0.0619 | 0.002** | 0.2260 | 0.001*** | 0.4595 | 0.001*** | 0.1216 | 0.001*** |
| Temperature | 0.1583 | 0.001*** | 0.2629 | 0.001*** | 0.6369 | 0.001*** | 0.1610 | 0.001*** |
| Precipitation | 0.1265 | 0.001*** | 0.1550 | 0.001*** | 0.0476 | 0.197 | 0.0174 | 0.189 |
| pH | 0.0095 | 0.257 | 0.0246 | 0.128 | 0.1176 | 0.012* | 0.0153 | 0.228 |
| EC | 0.0180 | 0.070 | 0.0592 | 0.007** | 0.0141 | 0.664 | 0.0094 | 0.372 |
| Sand content | 0.0106 | 0.241 | 0.0669 | 0.003** | 0.2813 | 0.001*** | 0.0439 | 0.014* |
| Clay content | 0.0162 | 0.093 | 0.0332 | 0.073 | 0.0479 | 0.188 | 0.0232 | 0.106 |
| SNC | 0.0172 | 0.088 | 0.0719 | 0.004** | 0.1334 | 0.009** | 0.0079 | 0.436 |
| SCC | 0.0142 | 0.119 | 0.0650 | 0.003** | 0.0122 | 0.654 | 0.0231 | 0.095 |
